# Limb Kinematics, Kinetics and Muscle Dynamics During the Sit-to-Stand Transition in Greyhounds

**DOI:** 10.3389/fbioe.2018.00162

**Published:** 2018-11-16

**Authors:** Richard G. Ellis, Jeffery W. Rankin, John R. Hutchinson

**Affiliations:** ^1^Structure & Motion Laboratory, Department of Comparative Biomedical Sciences, The Royal Veterinary College, North Mymms, United Kingdom; ^2^Pathokinesiology Laboratory, Rancho Los Amigos National Rehabilitation Center, Downey, CA, United States

**Keywords:** biomechanics, dog, static optimization, musculoskeletal system, muscle fascicle, opensim

## Abstract

Standing up from a prone position is a critical daily activity for animals: failing to do so effectively may cause an injurious fall or increase predation susceptibility. This sit-to-stand behaviour (StS) is biomechanically interesting because it necessitates transitioning through near-maximal joint motion ranges from a crouched (i.e., poor mechanical advantage) to a more upright posture. Such large joint excursions should require large length changes of muscle-tendon units. Here we integrate experimental and musculoskeletal simulation methods to quantify the joint motions, limb forces, and muscle fibre forces, activations and length changes during StS in an extreme athlete—the greyhound—which has large hindlimb muscles bearing short-fibred distal muscles and long tendons. Study results indicate that hindlimb anti-gravity muscle fibres operate near their ~50% limits of length change during StS; mostly by starting at highly lengthened positions. StS also requires high muscle activations (>50%), in part due to non-sagittal motions. Finally, StS movements require passive non-muscular support in the distal hindlimb where short-fibred muscles are incapable of sustaining StS themselves. Non-locomotor behaviours like StS likely impose important trade-offs between muscle fibre force capacity and length changes, as well as active and passive mechanisms of support, that have been neglected in locomotor biomechanics studies.

## Introduction

As a common activity of daily living, the ability to stand up (from lying down, a deep squat or chair) is critical to the welfare of humans and other species (e.g., Zannier-Tanner, 1965; Nickel et al., 1986; Janssen et al., 2002; Feeney et al., 2007). However, previous work on the sit-to-stand transition in humans (StS) indicates that the movement is one of the most biomechanically demanding activities—even more challenging in some ways than running or jumping (Rodosky et al., 1989; Yoshioka et al., 2009; Dall and Kerr, 2010; Fluit et al., 2014). Indeed, healthy adult humans may use over 80% of their maximum muscular effort for selected muscles (e.g., knee extensor torque) to rise from a seated position in a chair (Hughes et al., 1994, 1996; Gillette and Hartman, 2003; Bieryla et al., 2007; Savelberg et al., 2007). It is widely considered that, because of these demands, the way humans stand up changes during their lifetime from a more demanding pattern (“momentum transfer strategy”) to one that minimizes muscular effort in elderly humans with muscle weakness (“stabilization strategy”) (e.g., Savelberg et al., 2007; Van der heijden et al., 2009). Hence standing up is constrained by the strength-to-weight ratio in humans in some way, although the specific mechanisms and control strategies used to overcome strength-to-weight limitations during this movement remain controversial (Pandy et al., 1995; Bobbert et al., 2016; Actis et al., 2018; Shia et al., 2018), and neurophysiological control mechanisms for StS are still being deciphered (e.g., Silva et al., 2013).

Even though it is much less studied in non-human animals, StS capacity is used as a key marker for well-being in horses (Gardner, 2011) and cattle (Lidfors, 1989). Indeed, StS capacity appears to vary greatly between species: medium to large-sized mammalian carnivores tend to spend more time lying down and sit down or stand up more easily than herbivores (Nickel et al., 1986). Furthermore, the biomechanical demands associated with StS movements likely differ markedly between humans and non-human animals. For example, most human StS studies focus on rising from a chair (Pandy et al., 1995; Khemlani et al., 1999; Reisman et al., 2002; Buckley et al., 2009; Yoshioka et al., 2009; Fujimoto and Chou, 2012) whereas other animals mostly sit/lie on the ground. The more crouched starting position potentially requires larger joint moments at weaker strength-to-weight ratios due to low mechanical advantage (e.g., Hughes et al., 1996). Examination of StS in non-humans would therefore allow independent assessments of how body size, limb geometry or even ecology influence StS mechanics in ways not possible through exclusively human studies. Despite these potential benefits, biomechanical investigation of the StS transition remains almost non-existent in non-human animals (one exception: Feeney et al., 2007).

StS clearly imposes markedly different musculoskeletal demands compared to other common tasks such as walking or running. Larger animals locomote using relatively straight limbs and undergo only moderate ranges of joint motion. Further, the favourable limb orientation with respect to the ground reaction force (GRF) during locomotion helps reduce the joint moments necessary for body support and propulsion by increasing the effective mechanical advantage (Biewener, 1989). In contrast, StS begins from a flexed limb posture and covers a large range of joint motion, resulting in large GRF moment arms and thus presumably disadvantageous mechanical advantage. These substantial kinematic and kinetic differences between typical locomotion and the StS movement should impose divergent constraints on muscle-tendon unit architecture.

The evolution of muscle architecture has tended to match muscle anatomy to tasks, for example adapting fibre lengths and pennation angles to suit either long contractile lengths or high force-generation, especially in animals with cursorial (e.g., long-legged with shorter proximal segments and longer tendons such as a horse or dog) morphology (Sacks and Roy, 1982; Alexander and Ker, 1990; Payne et al., 2005; Smith et al., 2006; Williams et al., 2008). Musculotendon units seem especially well adapted to steady state locomotion, often operating at muscle lengths that place individual sarcomeres near their optimal length for force production (e.g., Dimery, 1985; Cutts, 1989; Burkholder and Lieber, 2001; Rubenson et al., 2012). This adaptation, however, may impose additional costs for executing other behaviours such as StS. In particular, the large ranges of joint motion and initially flexed limb postures in StS likely require large changes in muscle sarcomere lengths, causing muscles to operate over less optimal regions of their intrinsic force-length relationship and reducing their ability to generate forces to match the required joint moments (Zajac, 1989). Alternatively, tendons may be tuned to assist in reducing these major length changes during StS. Considering the intrinsic muscle force-length relationship, what range of muscle fibre lengths do animals use when completing StS? How do these length changes influence limb muscle activity and force production during the StS movement?

In animals, StS also commonly appears to involve substantial mediolateral movements such as rolling or joint add/abduction (Nickel et al., 1986). These non-sagittal motions may allow animals to partially circumvent the musculoskeletal constraints on sagittal plane movement imposed by locomotion, although this also may obviate the usage of adaptations to those sagittal motions. Such StS strategies may allow for the transfer of power produced by core muscles to the limb or the more effective use of muscles whose primary actions are non-parasagittal. Hip adduction, for example, may be less constrained by locomotion and might allow muscles to be better adapted to StS. How do animals use non-parasagittal muscle activity to meet StS movement demands? Given its markedly different task requirements from locomotion, StS provides a unique window into how animals deal with these biomechanical trade-offs.

Much as constraints on muscle fibre length changes may require animals to use unusual mechanisms during StS such as increased parasagittal movements or muscle activity, passive mechanisms of support might be used to a greater degree in StS than in locomotion. In addition to tendons, ligaments, joint capsules and other tissues (hard and soft) are plausible candidates for providing joint support in StS. For example, Rankin et al. (2016) showed that during walking and running, ostriches likely require substantial passive support (e.g., ligaments and/or bony stops) to resist hip abduction (see also Manafzadeh and Padian, 2018). In humans, resistive moments due to passive mechanisms have been recorded at extreme joint angles: the StS movement may use non-muscular tissues to support the motion (Davy and Audu, 1987). Similarly, such passive support may be used by other animals, including cursorial mammals.

The aim of this study was to characterize StS limb and muscle mechanics in an animal with cursorial limb morphology (i.e., greyhound, *Canis familiaris*). We chose to primarily examine the hindlimb movement as this would provide a better comparison with human data. We hypothesized that:

1. Hindlimb muscle lengths would operate near their functional limits (~50% shortening/lengthening of contractile units following conventional Hill model assumptions; Zajac, 1989; Millard et al., 2013) during StS.
2. StS would require relatively high muscle activations (>50%) in the key extensor muscles (due to the large range of motion in joint extension necessary) and hip adductors (resulting from non-parasagittal motions).
3. Because of large muscle fibre length changes and activations, passive support mechanisms (e.g., ligaments) would be critical for StS, especially distally in the hindlimb where short-fibred muscles might not be able to sustain StS themselves.

As a first step in answering these fundamental questions on mammalian musculoskeletal design and behaviour during StS, we measured the kinematics and kinetics of healthy adult greyhound dogs as they stood from a crouched, prone position. These data were used with a newly constructed, detailed musculoskeletal model of a greyhound hindlimb to estimate the muscle forces, activations and length changes required to reproduce measured StS dynamics.

We performed a series of static optimizations in which we varied the parameters of our model to simultaneously test the sensitivity of various modelling assumptions and the extent to which different musculoskeletal and movement factors may contribute to the StS movement. First, we assumed that tendons remained rigid (as classic experimental studies of dogs did; e.g., Goslow et al., 1981), which allowed us to test if muscle fibres alone could satisfy the demands associated with the measured StS dynamics (testing Hypotheses 1 and 3) because introducing tendon elasticity would likely decrease the need for muscular activity by allowing tendons to contribute to length change and force demands during StS. Second, we included non-sagittal motions in our optimization framework to test if hip adductor muscles were unusually challenged by the StS motion (Hypothesis 2). Last, we tested the effect of varying tendon slack length (TSL) on our output parameters. Varying TSL would necessarily change operating fibre length during StS (testing Hypothesis 1, and by extension Hypothesis 2) and, at least for MTUs with short fibres and long tendons, would change the need for passive support (represented in our modelling framework as “reserve actuators”; testing Hypothesis 3).

## Materials and methods

### Overview

Eight pure and half-breed greyhounds (5 males and 3 females; body mass 27 ± 5 kg, torso length 61.0 ± 7 cm; shoulder height 60.5 ± 8 cm; hip height 61.6 ± 3.9 cm; mean ± 1 *SD*) completed a total of 149 StS trials. In each trial, the dog stood up from lying down while we measured joint kinematics and ground reaction forces. Trials lasted 10 s, although each StS cycle took only a fraction of this time (Supplementary Video S1). Experiments were approved by the Ethics and Welfare Committee of the Royal Veterinary College (approval number URN 2012 1184). All standard biosecurity and safety procedures were followed. We also created a detailed musculoskeletal model of a greyhound hindlimb using commercial musculoskeletal modelling software (SIMM; Musculographics, Inc, CA, USA; Delp and Loan, 1995), which we then imported into OpenSim software (Stanford, CA, USA, Delp et al., 2007). OpenSim's static optimization routine was used with the model and experimental data to estimate muscle fibre lengths, activations, and forces during a representative StS motion. Experimental data, scan data, model and simulations are openly provided at https://figshare.com/projects/Limb_Kinematics_Kinetics_and_Muscle_Dynamics_During_the_Sit-to-Stand_Transition_in_Greyhounds_data/55904, which include details on all model parameters.

### Experimental data

**Figure 1:**
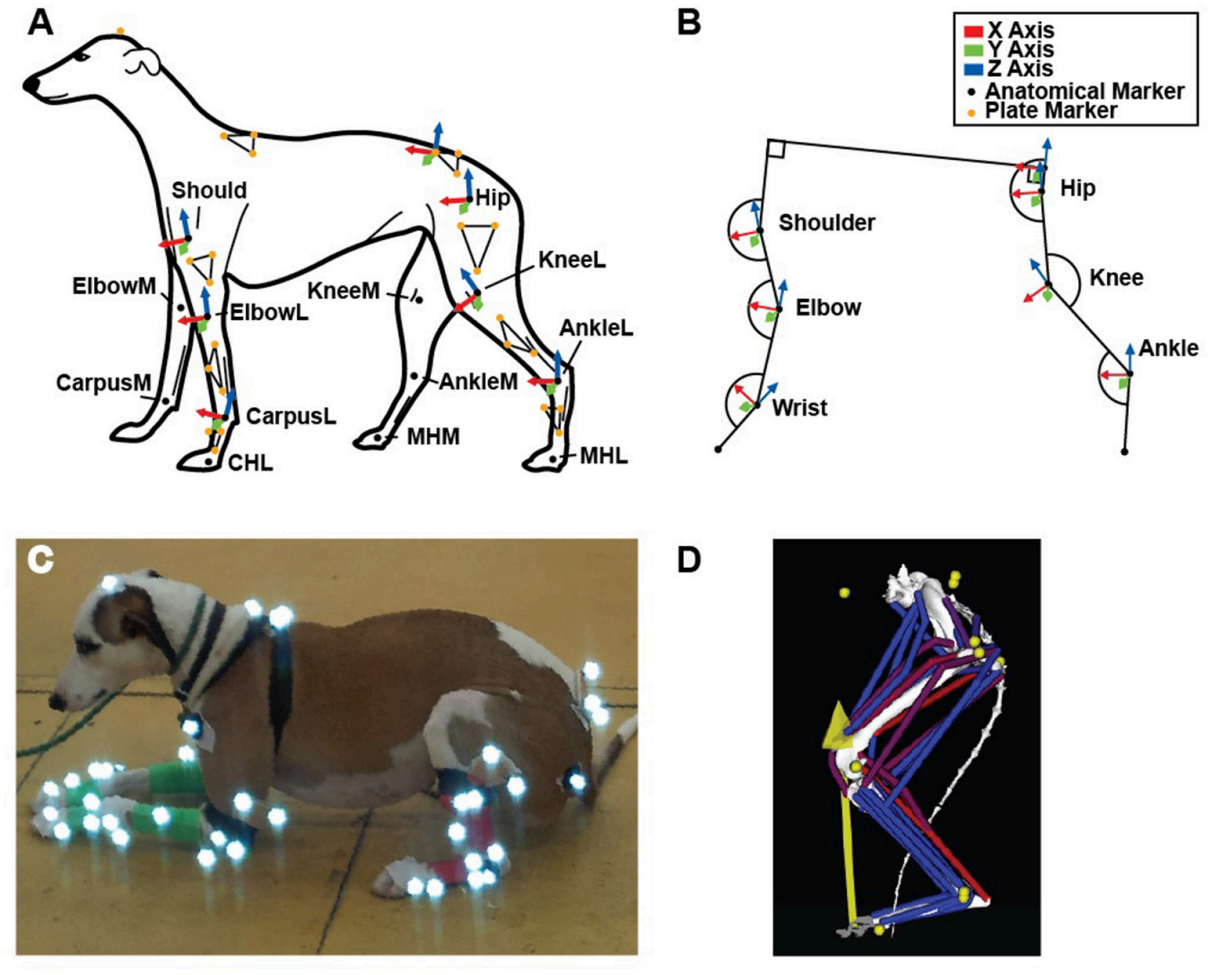
**(A)** Schematic of fore- and hindlimb marker locations and corresponding segment axes (Technical Coordinate System- see Methods) used to determine joint angles, in left lateral view. **(B)** Conventions for X, Y and Z axes and joint angle measurements (calculated from our technical and anatomical coordinate systems), corresponding to the position shown in **(A)**. **(C)** Example subject with marker set. **(D)** Complete musculoskeletal model, with marker and ground reaction forces represented by yellow circles and arrow, respectively.

To obtain limb joint angles during the StS movement, we affixed rigid three-marker clusters to each dog's back, pelvis and limb segments (14 clusters total) (Figure 1A). Additionally, we placed at least 17 individual infrared-reflective markers on anatomical landmarks to generate an Anatomical Coordinate System (ACS) (Table 1, Figures 1A–C). To improve the calculation of the ACS, additional markers were placed on the medial aspect of the elbow, knee, wrist, ankle and foot midway through the study, increasing the number of anatomical markers to 29 (Table 1). As a result, approximately half the trials were recorded with the original marker set and the other half with the expanded set. See Supplementary Text S1 for further information. In all trials, the dogs started in a symmetric, crouched position. Trials were deemed valid when the dog did not adjust its foot position (i.e., by stepping) during the StS movement. We focus here only on the (left) hindlimb data but for completeness and comparison, we provide experimental results from the forelimbs in the Supplementary Information (cited in Results below). In all cases, motion data were collected at 180 Hz using 12 Qualisys Oqus cameras (Qualisys AB, Gothenburg, SE).

**Table 1:** Experimental marker set used to define the anatomical coordinate systems (i.e., segment axes) used to calculate joint angles during the StS movement.

**Segment**	**Marker name**	**Anatomical location**
Humerus	Should	Midway between the spinous process of the scapula and the greater tubercle of the humerus
	ElbL	Lateral condyle of the humerus
	ElbM	Medial condyle of the humerus
Radius/Ulna	WristL	Styloid process of the ulna
	WristM	Styloid process of the radius
Carpals/Metacarpals/ Phalanges	MCHL	Head of metacarpal V
	MP4	Dorsally on the head of the middle phalanx of manus digit IV
Femur	Hip	Greater trochanter of the femur
	KneeL	Lateral condyle of the femur
	KneeM	Medial condyle of the femur
Tibia/fibula	AnkleL	Lateral malleolus of the ankle
	AnkleM	Medial malleolus of the ankle
Tarsals/Metatarsals/ Phalanges	MTHL	Head of metatarsal V
	MTHM	Head of metatarsal II

*The “Marker Name” column lists the markers shown in Figure 1A and Supplementary Text S1*.

Joint angles were calculated by first defining two coordinate systems for each segment as outlined in Supplementary Text S1. The rigid marker clusters (Technical Coordinate System [TCS]; Figure 1C) were related to the anatomical markers (ACS) using a static reference posture taken after a dog had completed StS and was standing normally. Marker data were filtered (4th order Butterworth, 6 Hz low pass), and then Cardan angles of rotation order Y X' Z” (Robertson et al., 2013) were calculated from the marker data using custom MatLab (The MathWorks, Natick, MA, USA) scripts. In this system, rotations about the Y-axis represented joint flexion/extension (Figure 1B), and increasing joint angles in the figures correspond to extension for all joints.

Ground reaction forces (GRF) and centre of pressure (CoP) location were collected at 1,800 Hz using two Kistler force platforms (Model 9268BA, Kistler Holding AG, Winterthur, CH). Dogs were positioned such that both forelimbs were on one force plate and both hindlimbs were on a second plate at the beginning of each trial. Force data were de-meaned, low-pass filtered (4th order Butterworth, 6 Hz), down-sampled to 180 Hz, and synchronized with the marker data.

In some trials, a few foot markers were not visible in the initially crouched posture at the beginning of StS, especially on the medial side of all limbs. However, because these limbs did not initially move during the motion, we filled the missing data using the first valid value. The motion data in trials for which this process was followed were consistent with results from more complete trials measured in this and a previous study (Feeney et al., 2007). Joint angles were calculated for each trial and outliers were excluded from further analysis when a joint's angle differed from the group average by more than ~45° (generally well outside of 1 S.D. from the group mean). Thus, a total of 21 forelimb trials (four dogs) and 39 hindlimb trials (six dogs) were then used for detailed analysis. These trials were time-normalized to the average trial duration across dogs (0.64–1.94s range; 1.14 ± 0.16 mean ± *SD*), resampled to 100 points per trial, and averaged first within a subject and then between subjects.

### Musculoskeletal model

We constructed a detailed musculoskeletal model of a greyhound hindlimb in SIMM (MusculoGraphics Inc., Santa Rosa, CA, USA; Delp and Loan, 1995; Thelen et al., 2003) directly from CT data. The model had 4 segments (pelvis, thigh, shank and foot) articulated by 3 joints (hip, knee and ankle) and 39 actuators representing hindlimb muscles.

We generated model joints, segments, and muscle paths from an adult greyhound's cadaveric left hindlimb (formalin-preserved; obtained from the Royal Veterinary College's teaching collection via a previous post-mortem donation), via our own dissections and image analysis. The hindlimb was CT scanned (GE Lightspeed 8-detector unit; 2.5 mm thick helical slices at 120 kVp and 100 mA; “boneplus” reconstruction algorithm; limb distal to the knee scanned at 0.625 mm slice thickness) and segmented (Mimics 15.01, Materialise, Leuven, Belgium; semi-automatic segmentation using default bone thresholds for bones, and manual segmentation for muscles) to create 3D objects for each major leg bone and muscle (Supplementary Video S2).

Model joints were defined by first manually aligning regular shapes (cylinders and spheres) to each joint's anatomical centres of rotation (3DS Max 2013, Autodesk, San Rafael, California, USA). The positions and orientations of these objects were then used to calculate joint centres and axis orientations (Allen et al., 2013). The entire model was free to rotate and translate relative to the global reference frame via a pelvis/torso segment. This segment was defined to move about a point on the midline between the two ilio-sacral joints. This location corresponded to one of our body markers and was used to define pelvis translations and rotations relative to the ground. The hip, knee and ankle joints (Figure 1B) were all initially allowed three degrees of rotational freedom in the model; toe joints were immobile.

For all joints, the X-axis pointed cranially (i.e., horizontally) in the original reference pose (limb fully straightened at 0° joint angles). The Y-axis was oriented to the animal's left (lateral) and the Z-axis was oriented vertically. Thus, overall, joint rotations were defined relative to the reference pose, and all axes were orthogonal. For example, knee ad/abduction was defined as the angle between the craniocaudal axes of the femur and tibia.

**Figure 2:**
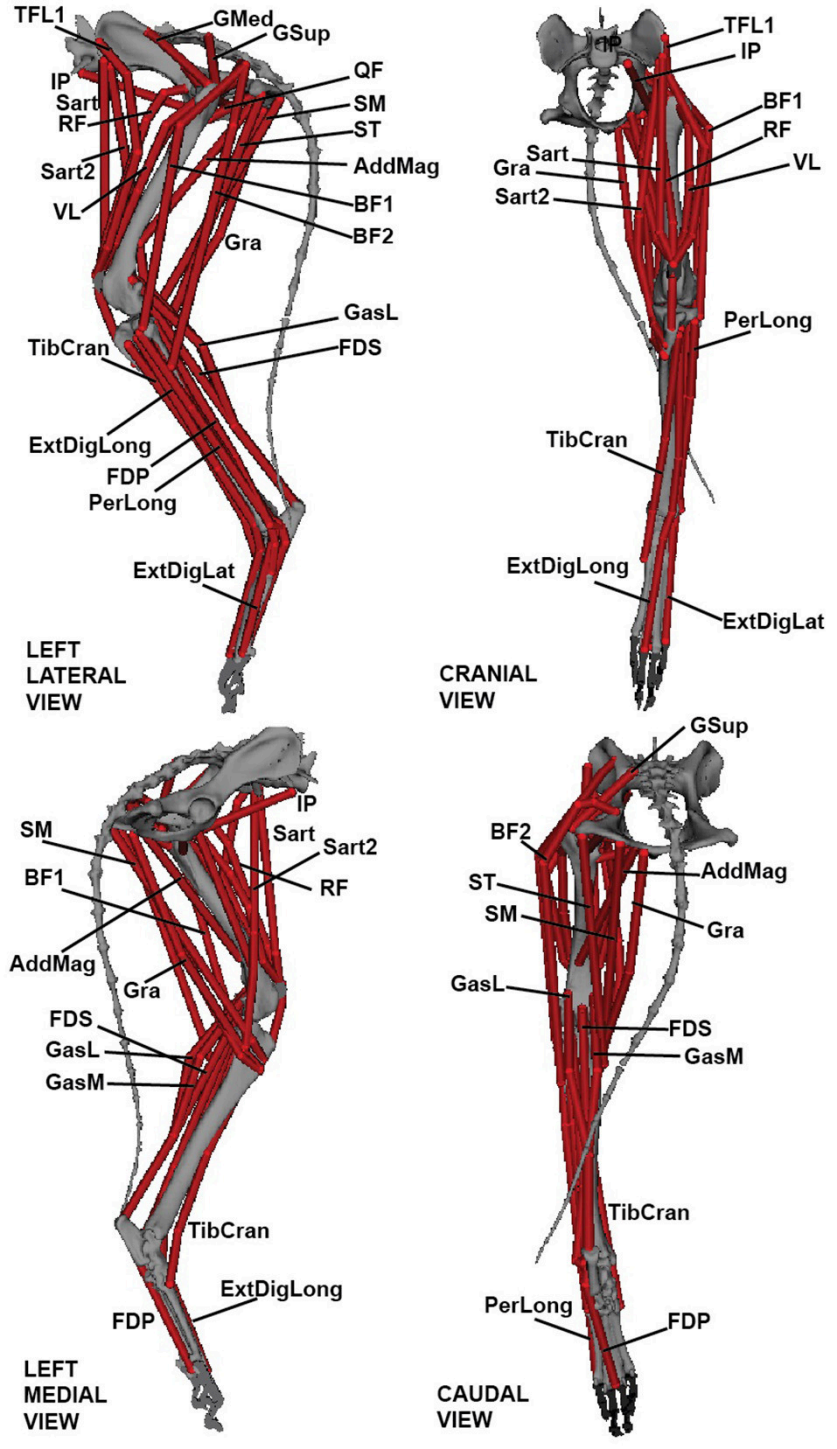
Three-dimensional greyhound hindlimb musculoskeletal model in lateral, cranial, medial, and caudal views. Muscle labels correspond to abbreviations in Table 2.

Segment and joint definitions were combined in SIMM software (MusculoGraphics Inc., Santa Rosa, CA, USA; Delp and Loan, 1995; Thelen et al., 2003) to create a three-joint musculoskeletal model of the left greyhound hindlimb; analogous to those of Pedersen et al. (1991), Shahar and Milgram (2001) and Helms et al. (2009) (see also Dries et al., 2016). A total of 39 musculotendon unit (MTU) actuators were implemented to represent the major hindlimb muscles (Figures 1D, 2). The MTU paths were defined by aligning each path through the centre of the corresponding segmented 3D muscle object (Supplementary Video S2) in SIMM. Joint angles were then varied throughout their range of motion and MTU paths and further adjusted to ensure plausible muscle paths throughout the entire joint range of motion. We re-examined each MTU path and length while the model moved through our measured kinematics and adjusted the MTU paths to ensure smooth transitions between the various wrapping surfaces and via points.

#### Mtu parameters

MTUs were defined using a Hill-type muscle model (Millard et al., 2013) that included intrinsic force-length-velocity relationships. To obtain the muscle parameters needed for our model, we measured muscle fascicle length (assumed to equal optimal fibre length), muscle mass and pennation angle by dissection. These are standard muscle architecture parameters, with physiological cross-sectional area allowing calculation of maximal force via a maximal isometric muscle stress of 300 kNm^−2^ (Table 2; see Sacks and Roy, 1982; Alexander and Ker, 1990; Payne et al., 2005; Smith et al., 2006; Williams et al., 2008). Next, we estimated tendon slack length (TSL) according to the approach described by Manal and Buchanan (2004). TSL is the length of the virtual tendon in the model at which passive force development is zero. A measured muscle fibre (i.e., fascicle) resting length was assumed to be equivalent to its optimal length for isometric force generation. Note that our simulations, as mandated by static optimizations in OpenSim, had rigid tendons but TSL input values were still required. Sensitivity analyses (below) addressed these TSL assumptions.

**Table 2:** Muscle architectural parameters measured from dissection of a greyhound hindlimb.

**Joint**	**Muscle**	**Abbreviation**	**Measured PCSA (cm^2^)**	**Pennation angle (°)**	**Fmax (N)**	**Fmax, (Williams et al., 2008) (N)**	**Ratio**	**Measured**		**Model**	
								**Optimal fibre length (cm)**	**Tendon slack length (cm)**	**Optimal fibre length (cm)**	**Tendon slack length (cm)**
Hip	Adductor brevis	AddBrev	3.49	7	103	770 (combined)		2.94	0.71	2.94	0.71
Hip	Adductor magnus	AddMag	14.11	0	423		0.68	12.50	1.67	12.50	1.67
Hip	Gluteus medius	GMed	24.86	10	723	650	1.11	4.00	3.60	4.00	3.60
Hip	Gluteus profundus	GInt	4.35	8	128	230	0.56	2.08	0.67	2.08	**0.71**
Hip	Gluteus superficialis	GSup	4.37	22	113	840	0.13	5.07	0.01	5.07	**1.40**
Hip	Iliopsoas	IP	8.76	12	252	n/a		6.45	5.00	6.45	5.00
Hip	Obturator externus	ObtExt	1.89	0	57	n/a		5.93	0.29	**5.01**	**0.01**
Hip	Obturator internus	ObtInt	3.54	0	106	n/a		2.27	1.27	2.27	**2.20**
Hip	Pectineus	Pect	4.54	14	128	n/a		1.78	14.21	1.78	**13.61**
Hip	Quadratus femoris	QF	1.44	0	43	n/a		5.06	0.07	5.06	0.07
Hip	Tensor fascia lata	TFL1	18.02	32	389	310	1.25	2.71	22.60	2.71	**23.55**
Hip/Knee	Biceps femoris anterior	BF2	11.44	0	343	960 (combined)		12.00	14.95	**12.20**	**18.95**
Hip/Knee	Biceps femoris posterior	BF1	9.32	0	279		0.65	12.00	16.49	12.00	**17.40**
Hip/Knee	Gracilis	Gra	15.65	0	470	870	0.54	6.06	19.40	6.06	**19.40**
Hip/Knee	Rectus femoris	RF	12.20	12	350	790	0.44	3.98	23.39	**5.50**	**21.35**
Hip/Knee	Sartorius caudalis	Sart	2.39	0	72	310 (combined)		20.00	4.40	20.00	**5.80**
Hip/Knee	Sartorius cranialis	Sart2	0.57	0	17		0.29	24.65	0.05	24.65	**0.60**
Hip/Knee	Semimembranosus	SM	7.62	0	229	240	0.95	21.26	1.06	21.26	1.06
Hip/Knee	Semitendinosus	ST	5.41	0	162	360	0.45	18.34	6.46	18.34	6.46
Knee	Vastus lateralis & intermedius	VL	12.22	13	348	550	0.63	7.86	17.57	7.86	17.57
Knee	Vastus medialis	VM	6.66	14	188	770	0.24	8.63	17.12	8.63	17.12
Knee/Ankle	Flexor digitorum superficialis	FDS	37.16	28	864	1260	0.69	1.16	25.27	**4.66**	**21.36**
Knee/Ankle	Gastrocnemius lateralis	GasL	18.07	26	439	680	0.65	1.29	26.20	**3.09**	**24.40**
Knee/Ankle	Gastrocnemius medialis	GasM	20.30	27	486	610	0.80	1.85	26.50	**2.20**	**26.15**
Ankle	Extensor digitorum lateralis	ExtDigLat	0.83	10	24	n/a		1.28	34.68	1.28	**34.41**
Ankle	Extensor digitorum longus	ExtDigLong	2.94	0	88	155	0.57	5.14	30.60	5.14	30.60
Ankle	Flexor digitorum profundus	FDP	24.99	31	551	630	0.87	1.54	37.24	1.54	**36.59**
Ankle	Peroneus longus	PerLong	2.00	0	60	280	0.21	2.45	25.54	2.45	**24.92**
Ankle	Tibialis cranialis	TibCran	1.90	5	57	120	0.47	10.80	12.68	10.80	12.68

*The “Abbreviation” column lists the shortened names for muscles used in the main text and figures; all depicted in Figure 2. PCSA is physiological cross-sectional area, Fmax is maximal isometric force (also compared with mean data from Williams et al., 2008), “Ratio” indicates the ratio of our Fmax to that of Williams et al. (2008), and the “Measured” vs. “Model” columns show the original (from dissection) and modified (to keep muscle fibre lengths between 0.5 to 1.5 times optimal throughout StS) values of fibre (i.e., fascicle) and tendon slack lengths. Values where model and measured parameters differ are in bold font*.

When necessary, tendon slack lengths (TSLs) were further tuned so that normalized fascicle length was between 0.8 and 1.2 in the final posture at the end of each trial (i.e., in the normal standing posture; see Hicks et al., 2015). Fifteen of 29 muscles were adjusted in this way. For the gluteus superficialis, the method of Manal and Buchanan (2004) calculated a TSL of 0.1 mm, which was lengthened to 1.4 mm. With a total MTU length of 5.07 mm, this new length seemed physiologically more plausible than the calculated TSL of 0.1 mm but nevertheless resulted in a very large percentage change. We therefore present our changes in TSL length as a portion of total MTU length. On average, TSL was adjusted by 7.5% (*SD* 8.5%) of MTU length in these 15 muscles (Table 2).

Next, if muscle fibre lengths were still unrealistic (i.e., < 0.5 or >1.5 times resting length) throughout the StS motion, we increased (decreased) optimal fibre length while correspondingly shortening (lengthening) TSL (i.e., maintaining a constant MTU length) to ensure that all muscles operated between 0.5 and 1.5 times their optimal length (note this is related to our Hypothesis 1 and follows from fundamental principles of the Hill model of muscle; e.g., Zajac, 1989). This was done for 6 of 29 muscles. Muscles with this feature tended to have very short fibre lengths and comparatively long tendons (e.g., FDS fibre length increased from 1.16 to 4.66 cm with overall MTU length of 26.4 cm). On average, fibre length was adjusted by 7.0% (*SD* 6.0%) of MTU length. Together, these adjustments were sufficient for all muscles except for the ObtExt, where the initial shortening of the TSL was insufficient to achieve the target resting length. For this muscle, we therefore further decreased TSL to 0.1 mm and then also slightly decreased fibre length to stay within the target length throughout the movement (Table 2).

#### Model dimensions and scaling

The complete cadaver associated with the hindlimb used to construct our model was not available and no experimental subjects were (or could be) euthanized and scanned for our analysis. Thus, body segment dimensions were estimated as follows. Limb segment masses and moments of inertia were calculated for the thigh, shank and foot using a ray projection method to estimate the volume of each segment (Allen et al., 2013), assuming a uniform density of 1,060 kg/m^3^. To obtain more accurate body and head masses and moments of inertia for our model, we performed a linear regression of leg length against known body mass and body length for the eight experimental dogs (Supplementary Figure S1). These regression equations were used to estimate whole-body mass and length for the model animal. Regression equations derived by Amit et al. (2009) were then used to estimate torso and head masses and moments of inertia from the whole-dog dimensions. Our modelled greyhound hindlimb was 8.4% of estimated body mass, somewhat larger than that (6.79 ± 0.73% of body mass) found by Amit et al. (2009) using mixed breed dogs, but realistic in terms of the larger hindlimb segments in greyhounds (~8.7% body mass; see Colborne et al., 2005: Table 1). Furthermore, our estimates are reasonable matches to those of Williams et al. (2009; Table A1) although again of slightly lower mass (68–78% of their values).

The complete greyhound hindlimb model was imported from SIMM into OpenSim (Delp et al., 2007) for subsequent simulation and analysis. Because our kinematics and dissection data were from different animals, we first created a subject-specific model by scaling the model to be the same size as the animal associated with the representative trial using OpenSim's scale tool (Hicks et al., 2015). To do this, we measured the segment lengths of our experimental animals using the external joint markers (Figure 1A). We defined femur length as from the greater trochanter (Hip) to the lateral condyle of the knee (KneeL) and shank length as from the lateral epicondyle of the femur (KneeL) to the lateral malleolus of the tibia (AnkleL). We defined foot length as from the lateral malleolus of the tibia (AnkleL) to the head of the fifth metatarsal (MTHL). We then placed virtual markers (Table 1) at these same locations on our model and used them to scale the model to the experimental subject. Because the cadaver's torso was too incomplete to allow accurate placement of virtual markers, we used the average of the scale factors for the femur, shank and foot to scale the pelvis. Scale factors used are in Supplementary Text S1.

## Representative trial selection

**Figure 3:**
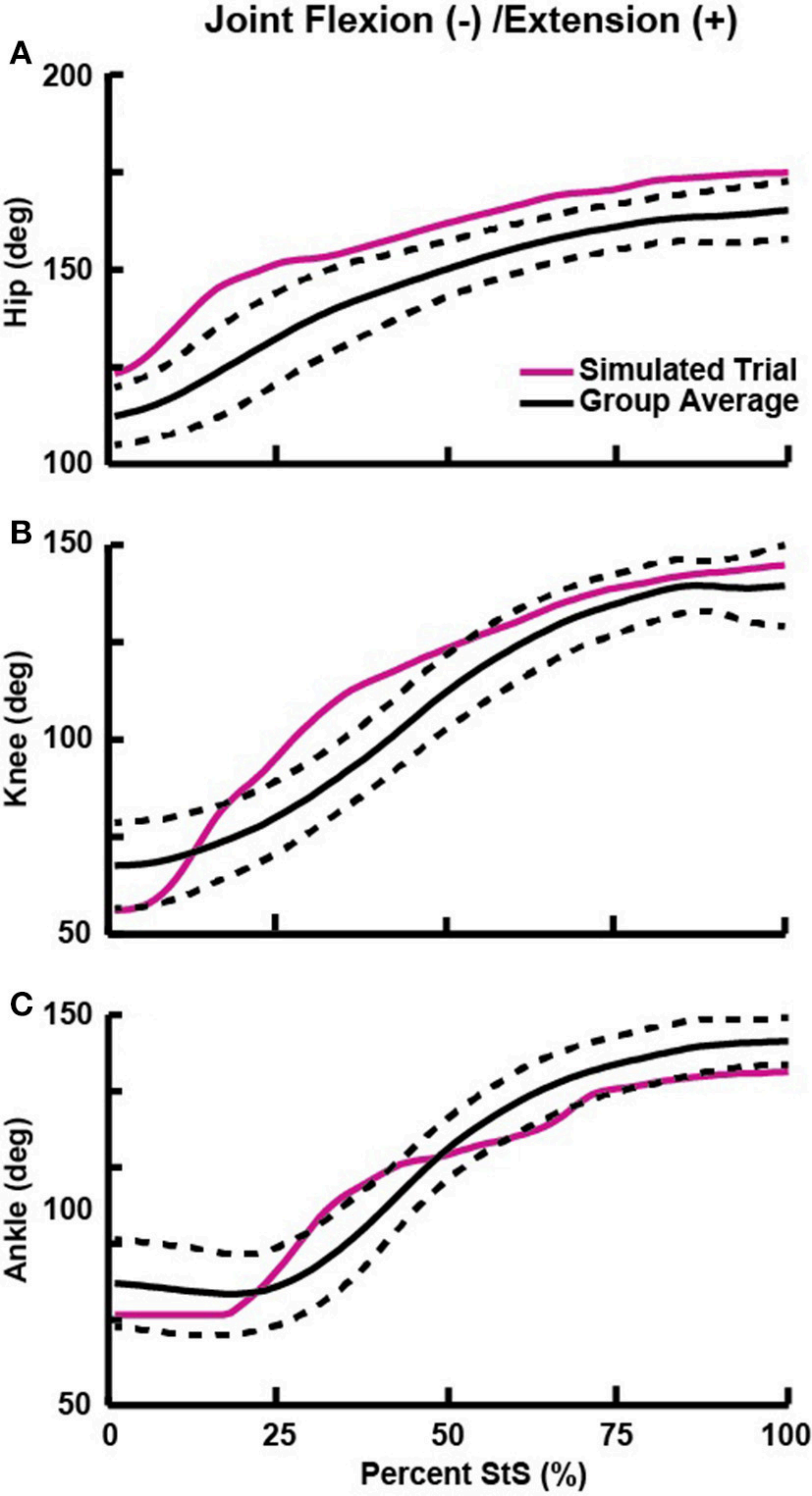
Flexion (−) and extension (+) angles for the hip **(A)**, knee **(B)**, and ankle **(C)** joints vs. percent StS cycle. Solid black line: group average kinematics. Dotted black lines: ± 1 standard deviation. Solid red line: Joint trajectory of the representative trial used for simulation (see also Table 3). Supplementary Figures S2, S3 show all data for non-sagittal joint motions.

**Figure 4:**
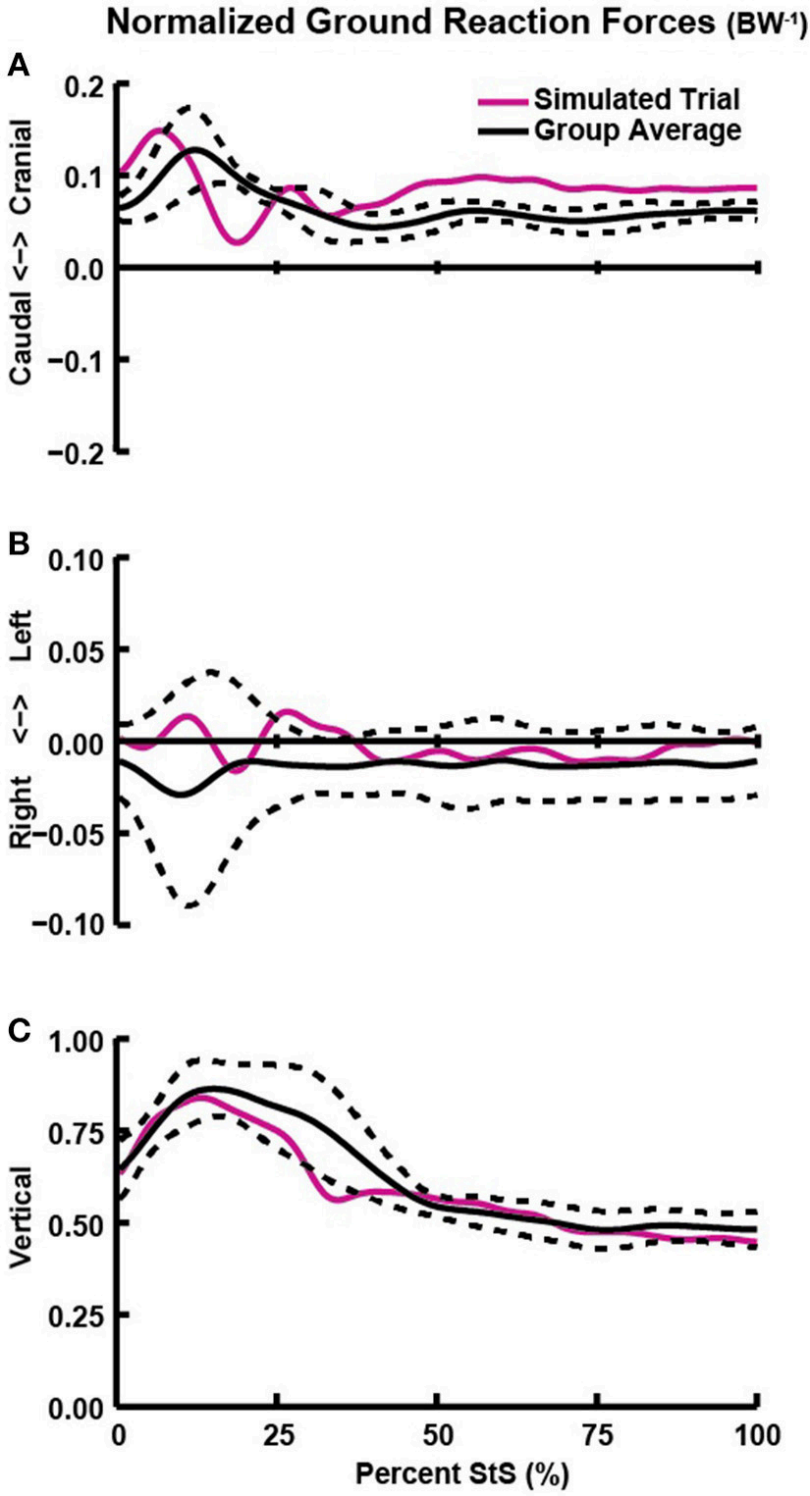
Hindlimb ground reaction forces (GRFs; normalized by body weight; BW^−1^) in the craniocaudal **(A)**, mediolateral **(B)**, and vertical/dorsoventral **(C)** directions vs. percent StS. Solid black line: group averaged kinetics. Dotted black line: ± 1 standard deviation. Solid red line: GRFs of the representative trial used for simulation.

We examined only trials where the dog's two fore- and hindfeet were entirely on their respective forceplates, creating a pool of 16 potentially useful trials. We animated our model using each of these trials and chose the best representative trial (Supplementary Video S3) by qualitatively assessing which had the most natural movement and had data within the range of observed kinematics and kinetics (Table 3, Figures 3, 4). Initially, we fixed knee and ankle add/abduction and int/external rotations to zero to reflect the limited movements possible at these joints (Newton and Nunamaker, 1985). However, the original non-zeroed data are shown in Supplementary Figures S2, S3 because we considered them in later sensitivity analyses.

**Table 3:** Measured range of motion for each greyhound hindlimb joint during StS.

	**Flexion/Extension**				**Add/Abduction**				**Internal/External Rotation**			
**Joint**	**Mean**	**±**	**1 S.D**.	**Trial**	**Mean**	**±**	**1 S.D**.	**Trial**	**Mean**	**±**	**1 S.D**.	**Trial**
Hip	53.4	±	5.6	51.5	18.5	±	9.9	21.8	8.7	±	4.3	**4.3**
Knee	78.5	±	10.7	88.6	30.2	±	10.2	29.1	25.7	±	4.9	21.2
Ankle	88.7	±	15.7	80.2	23.5	±	13.3	16.4	51.3	±	13.7	**15.9**

*Mean and standard deviation (SD) values were calculated using 39 trials from 6 dogs. “Trial” columns indicate the values for the representative experimental trial used for analysis in the study with values lying outside 1 SD of the mean emphasized in bold font*.

We measured only combined hindlimb GRF and centre of pressure (CoP) data from dogs during StS. Our pilot studies indicated that the dogs applied nearly vertical forces with each limb pair while standing up with minimal mediolateral force (Figure 4). In order to calculate single hindlimb GRFs and CoPs in our model, we assumed a zero mediolateral GRF and, assuming symmetry, divided measured vertical and craniocaudal GRFs by two. Next, we calculated the craniocaudal portion of the CoP movement (Supplementary Figure S4) and the mediolateral component of foot marker movement to create a composite, single hindlimb GRF dataset. Finally, we constrained the CoP to move along the midline of the model foot.

## Inverse dynamics and static optimization

**Figure 5:**
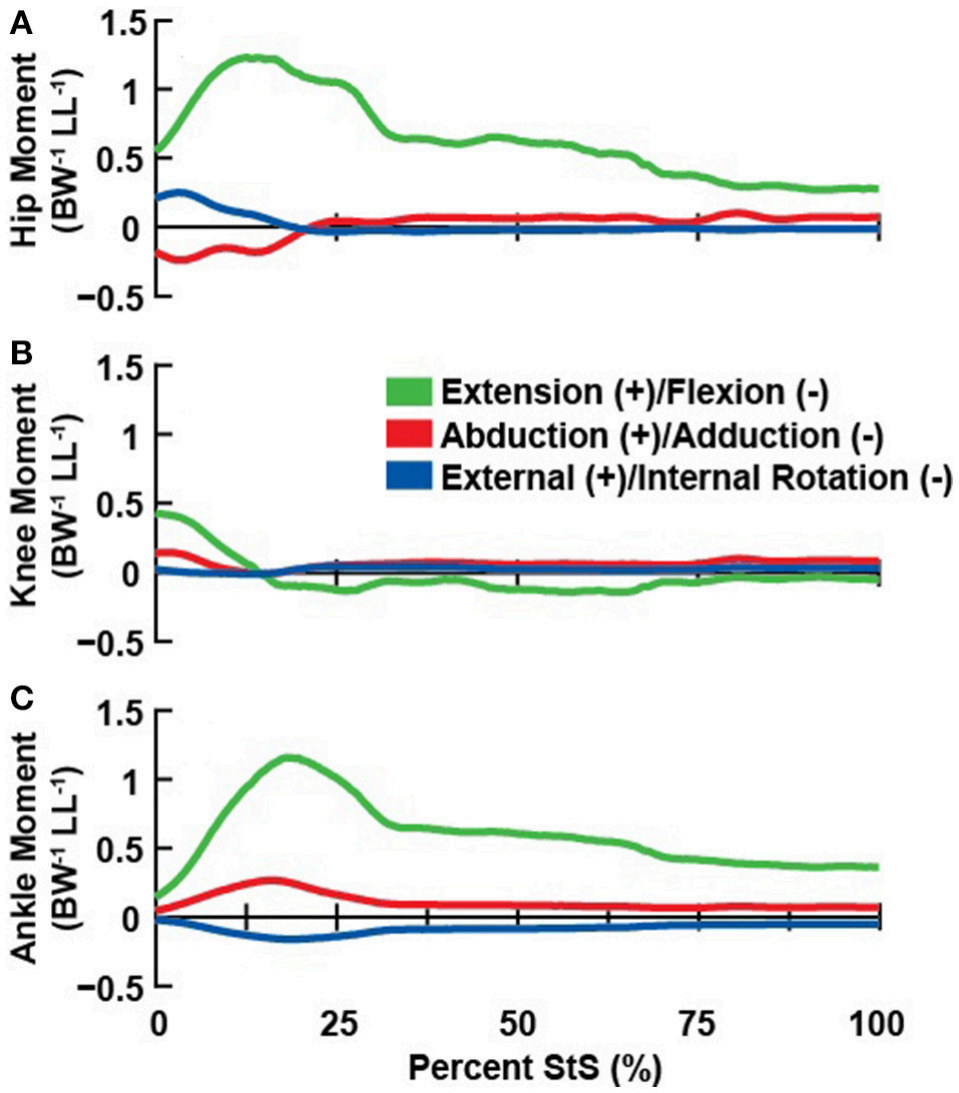
Net joint moments (normalized body weight times hindlimb leg length; BW^−1^ LL^−1^) about the hip **(A)**, knee **(B)**, and ankle **(C)** associated with the representative StS movement.

The inputs into OpenSim's analysis routines were (1) our hindlimb musculoskeletal model, (2) the hindlimb joint kinematics for our selected experimental trial and, (3) the hindlimb GRFs and CoP for this same trial. We calculated hindlimb joint moments using OpenSim's inverse dynamics routine (joint angle data low-pass filtered at 6 Hz using a 3rd order IIR Butterworth). Results were then transformed so that extensor, abductor and external rotation moments were defined to have a positive sign for all joints (Figure 5). Where useful for comparisons, forces were non-dimensionalized/normalized by dividing by body weight and moments by dividing by body weight (BW) times leg length (LL).

**Figure 6:**
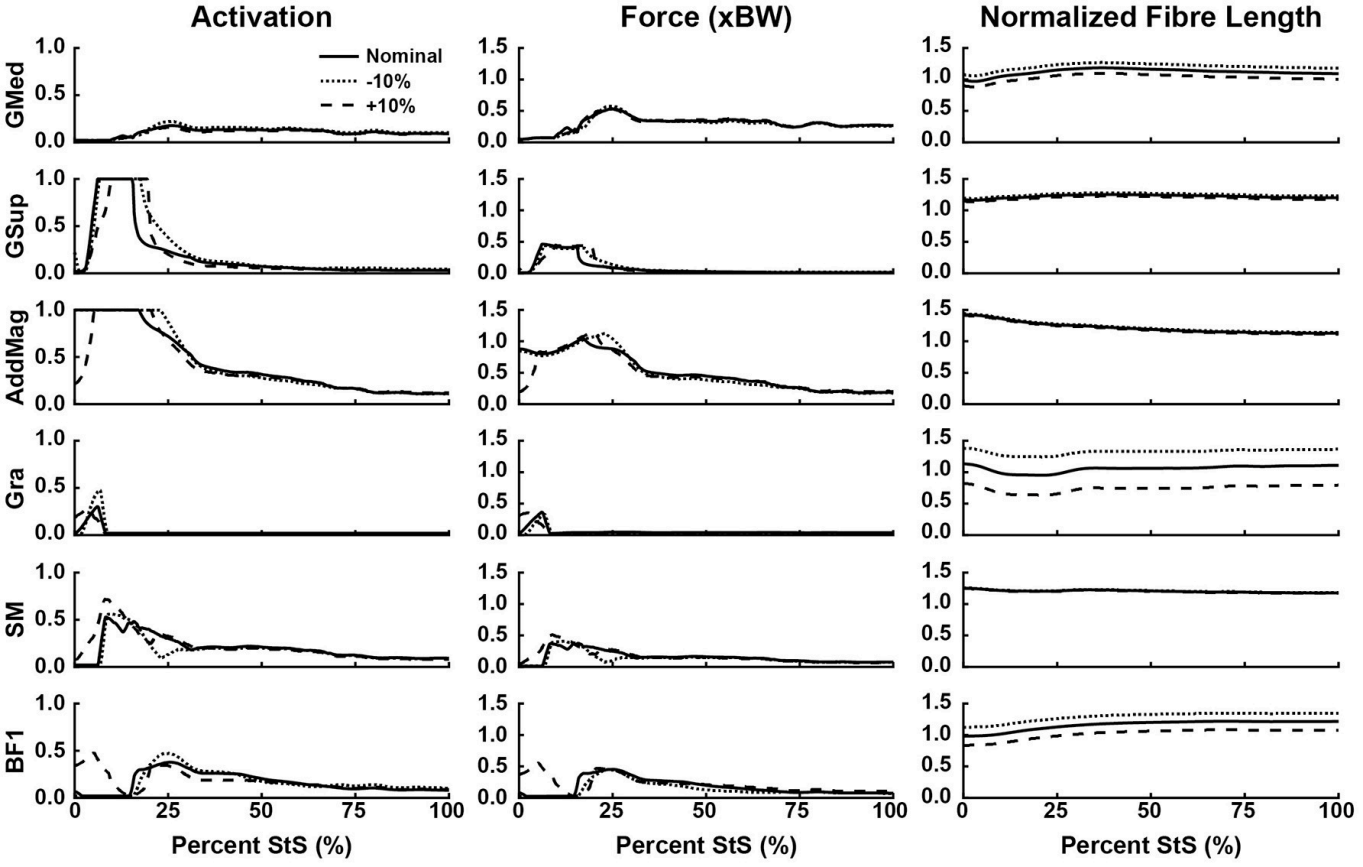
Simulated muscle activation, body weight-normalized muscle force (“xBW”), and normalized fibre length of the proximal thigh muscles (hip extensors and knee flexors, etc.) during StS. Only muscles where activation exceeded 20% of maximum are shown. Solid line: Nominal simulation. Dotted and Dashed lines: simulation results using altered tendon slack lengths (± 10% of nominal). Muscle abbreviations are defined in Table 2 and Figure 2.

**Figure 7:**
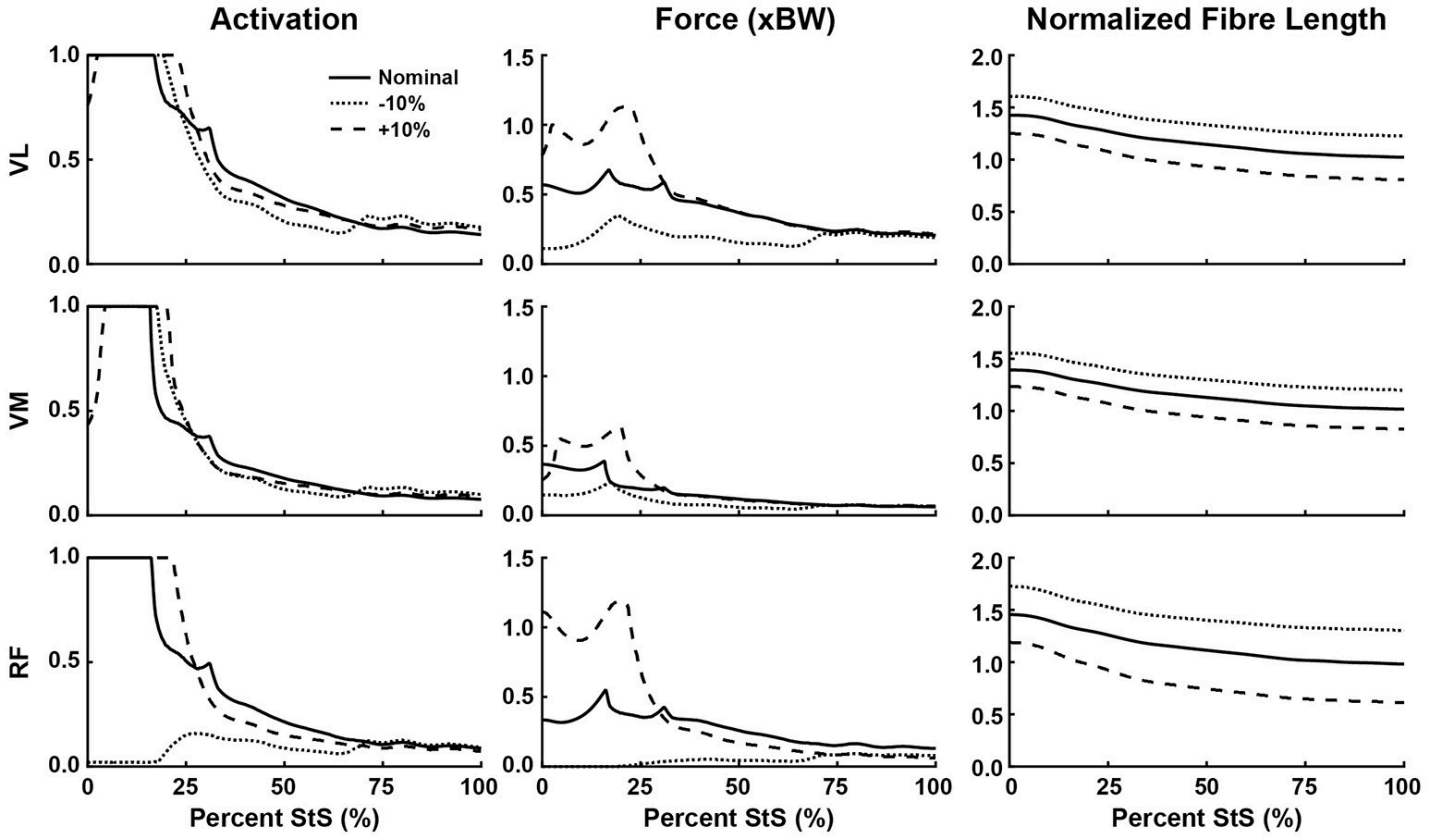
Simulated muscle activation, body weight-normalized muscle force (“xBW”), and normalized fibre length of the knee extensor muscles during StS. Only muscles where activation exceeded 20% of maximum are shown. Solid line: Nominal simulation. Dotted and Dashed lines: simulation results using altered tendon slack lengths (± 10% of nominal). Muscle abbreviations are defined in Table 2 and Figure 2.

**Figure 8:**
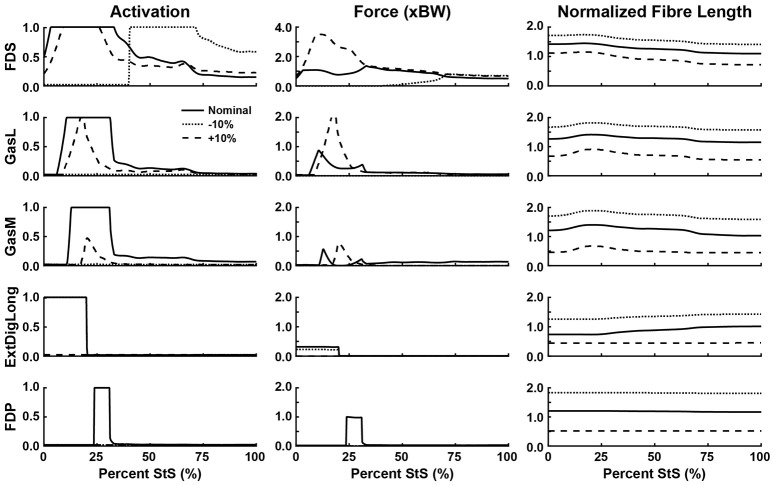
Simulated muscle activation, body weight-normalized muscle force (“xBW”), and normalized fibre length of the ankle and digital extensor muscles. Only muscles where activation exceeded 20% of maximum are shown. Solid line: Nominal simulation. Dotted and Dashed lines: simulation results using altered tendon slack lengths (± 10% of nominal). Muscle abbreviations are defined in Table 2 and Figure 2. Note the change in scale in “Force (xBW)” for FDS.

We then used OpenSim's static optimization routine to estimate muscle activation over the entire StS movement based on an objective function that minimized the summed square of muscle activations (Erdemir et al., 2007). This optimization routine incorporates a muscle's active force-length relationship but assumes tendons are inextensible and muscles do not generate passive forces. Only muscles with activations >20% of maximum or which applied >20% of body weight in force were examined further. A total of 14 out of 29 hindlimb muscles satisfied these criteria (Figures 6–8).

Additional “reserve actuator” torques were used at each joint to supplement the muscle-generated moments, with the optimization routine designed to only use these actuators when muscle activity alone was insufficient to generate the joint torques required to execute the movement. These reserve actuator torques were quantified during the analysis as it was conceivable that (our Hypothesis 3) muscles might be too weak to execute StS dynamics given the large potential muscle fibre length changes. Thus reserve actuators (approximating passive soft tissues etc.; Hicks et al., 2015; Rankin et al., 2016) might be “activated” to ensure that the StS was completed by the simulation. Furthermore, because the total forces acting on the torso would come from all four limbs simultaneously and not just the single hindlimb modelled here, “residual” forces and torques were applied to the pelvis segment and allowed to vary freely (Hicks et al., 2015).

## Sensitivity analyses

We performed a series of sensitivity analyses to test our major modelling and kinematic assumptions. We tested the robustness of our simulation results by first varying TSL by ± 10% (Scovil and Ronsky, 2006; Redl et al., 2007) and comparing how these TSL changes altered muscle activations, forces, fibre length changes, and reserve actuator torques. To test our kinematic data, we re-ran our simulation without constraining knee and ankle motion (i.e., treating our admittedly implausible three-dimensional kinematics for those joints as real; Table 3, Supplementary Figures S2, S3). This procedure provided an extreme set of model inputs that bracketed our conservative estimates of zero non-sagittal joint motions (i.e., to address part of our Hypothesis 2). Finally, we performed a static optimization using an additional trial from the same dog as an input (Supplementary Figures S6–S8).

## Results

We present our findings for StS in greyhounds starting with the experimental kinematics, followed by limb kinetics, then MTU dynamics and activation from simulations. After this, we present the results of the sensitivity analyses and reserve actuator contributions.

### Kinematics

The average StS duration for our experimental greyhound subjects was 1.14 ± 0.16 s (mean ± 1 *SD*). We found that hindlimb joint extension generally proceeded along a proximal to distal timing gradient, with hip extension occurring mostly from ~0 to 20% StS, knee extension occurring from ~10 to 50% StS and ankle extension occurring from ~25 to 60% StS (Figure 3). We observed substantial extension (~53–89°) at each hindlimb joint; more so than in the forelimbs (Table 3, Supplementary Table S1, Figure 3, Supplementary Figure S9). By comparison, the greyhounds used only relatively small add/abduction and int/external rotations (~9–30°) at five of the six measured joints (Supplementary Figures S2, S3). Our first and second datasets with different marker sets produced broadly similar flexion/extension motions but some divergent non-sagittal motions (Supplementary Text S1, Supplementary Figure S10).

The major exception to these small non-sagittal motions was the ankle joint, for which our data indicated a mean of ~51° of internal rotation (Table 3, Supplementary Figure S2). We suspected that the large ankle internal rotation was primarily due to how we constructed our model's joints, because the anatomical landmarks that we used to define rotation axes did not match their plausible rotational axes. A more thorough procedure (e.g., Rubenson et al., 2007) would improve accuracy, but would require collection of new experimental data (ideally using biplanar fluoroscopy to remove skin motion artifacts) and re-analysis of our simulations.

Regardless, we selected a representative trial with limited non-parasagittal motions (~16° internal rotation for the ankle; ~4–29° for other rotations; Table 3, Supplementary Figures S2, S3). This representative trial selected for initial simulation and further analyses had joint angles that generally fell within ± 1 *SD* of our broader experimental dataset (Figure 3, Supplementary Figures S2, S3), except as noted in Table 3. Additionally, these non-parasagittal motions were set to 0° in the nominal optimization.

### Kinetics

The external forces (GRFs) required for StS were exerted principally during the first 40% of StS (Figure 4). Dogs applied a small craniad force with their hindlimbs at ~15% of StS, initiating forward movement (Figure 4A). Mediolateral forces remained near zero throughout StS, reflecting the symmetric sit and stand positions imposed by our protocol (Figure 4B). Hindlimb vertical GRF was greatest (approaching 1x BW) at ~20% of StS (Figure 4C). Supplementary Text S1 describes the forelimb GRFs.

Our inverse dynamics analysis indicated the need for large net extensor moments about the hip and ankle joints (peaking at >1 normalized unit at ~15% StS), particularly early in StS (Figure 5) when the hindlimb was strongly flexed. Hip and ankle extensor moments remained near 0.5 normalized values for the hip and ankle throughout StS. In contrast, the knee joint required a brief extensor moment at the initiation of StS, (< 0.5 normalized moment) and then fell to near zero (small flexor net moment). We found only minimal non-sagittal moments for our representative trial, which peaked briefly (particularly for the ankle joint's internal rotator and abductor net moments; also external rotator and adductor moments around the hip) when extensor moments peaked, and then the non-sagittal moments fell to near zero. These moments changed partly due to a ~0.4 m craniad translation of the CoP (Supplementary Figure S4), enabled by the strongly plantigrade initial foot posture of the greyhounds early in StS.

### Muscle activations, forces and length changes

Consistent with the joint moment patterns (Figure 5), most estimated muscle activations and forces peaked by ~25% of the StS cycle (Figures 6–8, Supplementary Figure S5). We focus here on results from our nominal (i.e., original/representative) optimization (solid lines in Figures 6–8) and discuss sensitivity analyses of the effects of modifying TSL (dotted/dashed lines) later.

Simulated muscle activations reached the maximal possible value for two muscles acting around the hip (Figure 6): M. gluteus superficialis (GSup) and M. adductor magnus (AddMag), three major knee extensor muscles (Figure 7): M. vastus lateralis and medialis (VL and VM) and M. rectus femoris (RF), and five muscles acting around the ankle/toes (Figure 8): M. flexor digitorum superficialis (FDS), M. gastrocnemius lateralis and medialis (GasL and GasM), M. extensor digitorum longus (ExtDigLong) and M. flexor digitorum profundus (FDP). Intriguingly, some small, short-fibred hip muscles also showed brief pulses of ~1.0 activation early in StS (Supplementary Figure S5): M. obturator externus and internus (ObtExt and ObtInt), M. adductor brevis (AddBrev) and M. quadratus femoris (QF). The optimization predicted most other muscles to have low activations (< 0.5 peak values; e.g., Figure 6). Overall muscle activity decreased dramatically following the initial high activation, with a few muscles maintaining activations of ~0.1 by the end of StS (M. gluteus medius (GMed), AddMag, M. semimembranosus (SM), M. biceps femoris part 1 (BF1), the knee extensors, FDS and (to a lesser degree) GasL and GasM).

Several muscles developed large forces, approaching or exceeding body weight (1x BW; Figures 6–8). The FDS stood out in that regard, reaching a maximum force of ~1.5x BW, while the AddMag, GasL and FDP reached maximal forces of ~1.0x BW. The knee extensors (e.g., VM) barely exceeded 0.5x BW peak forces. The small hip muscles that had strikingly high activations early in StS only developed modest peak forces (~0.3x BW or less; Supplementary Figure S5). The largest absolute forces (Figures 6–8, Supplementary Figure S5) were developed by the AddMag (246 N), the FDS (321 N), and FDP (236 N) although >100N peak forces were predicted for GMed, GSup, BF1, RF, VL, and GasM. We observed slightly smaller peak forces for VM and GasL, and ExtDigLong exerted a maximum of 74 N.

We found that the AddMag, knee extensors (VL, VM, and RF) and FDS muscles were at extremely long fascicle lengths early in StS (~1.5 times optimal or resting length; Figures 6–8). Other muscles remained at lengths closer to optimal for force generation; e.g., the ExtDigLong and FDP stayed near resting length (Figure 8). At the hip (Figure 6), muscles such as the GMed and M. gracilis (Gra) shortened, lengthened, and then remained static, whereas the GSup and BF1 uniformly lengthened and the SM briefly shortened and then remained lengthened throughout StS. The knee extensors (Figure 7) consistently shortened, especially during early StS, concordant with the measured kinematics. The gastrocnemius muscles (GasL, GasM; Figure 8) showed an initial lengthening (approaching 1.5 times optimal length) very early in StS. The FDS also initially lengthened, following a similar pattern to the gastrocnemius muscles. All three muscles then gradually shortened.

### Sensitivity analyses

Our optimization results were sensitive to ± 10% changes in TSL for many muscles (Figures 6–8, dashed and dotted lines). The hip muscles were broadly less sensitive (Figure 6, Supplementary Figure S5), although activations and forces early in StS were affected, and normalized fibre length patterns for the Gra (especially) and BF1 (moderately) were shifted closer to the limit of 1.5 times optimal length (for shorter tendons) or toward/below optimal length (for longer tendons). The knee extensors (Figure 7) showed modest sensitivity of their activation patterns, except for the RF which dropped to very low activation if its tendon was shortened by 10%. Knee extensor activation was at 1.0 in early StS, with longer TSLs producing higher forces despite identical activations.

There were many substantial changes in muscle activation, force and length at the ankle (and toes) (Figure 8): with a shortened tendon, the FDS switched to purely late StS activation, whereas all of the other ankle/toe muscles dropped to 0 activation. Indeed, if the tendon was lengthened by 10% the ExtDigLong and FDP also eliminated their activity entirely, whereas FDS only declined moderately, GasL more so, and GasM strongly. Force patterns also changed in complex ways. The FDS produced more force (approaching 4x BW) early in StS with a longer tendon, or more (but only ~1x BW) late in StS with a shorter tendon. The GasL and GasM also showed greater forces with longer tendons early in StS, whereas the ExtDigLong had ~0 N force with a longer tendon or only a moderately reduced force (vs. nominal condition) with a shorter tendon; and the FDP had ~0 N force with any of the tendon changes. These results were reflected in the normalized fibre lengths in the same ways as for proximal muscles (shorter tendons leading to longer fibre lengths). The FDP was unusual in not changing length noticeably throughout the nominal simulation or with altered TSLs (Figure 8).

**Figure 9:**
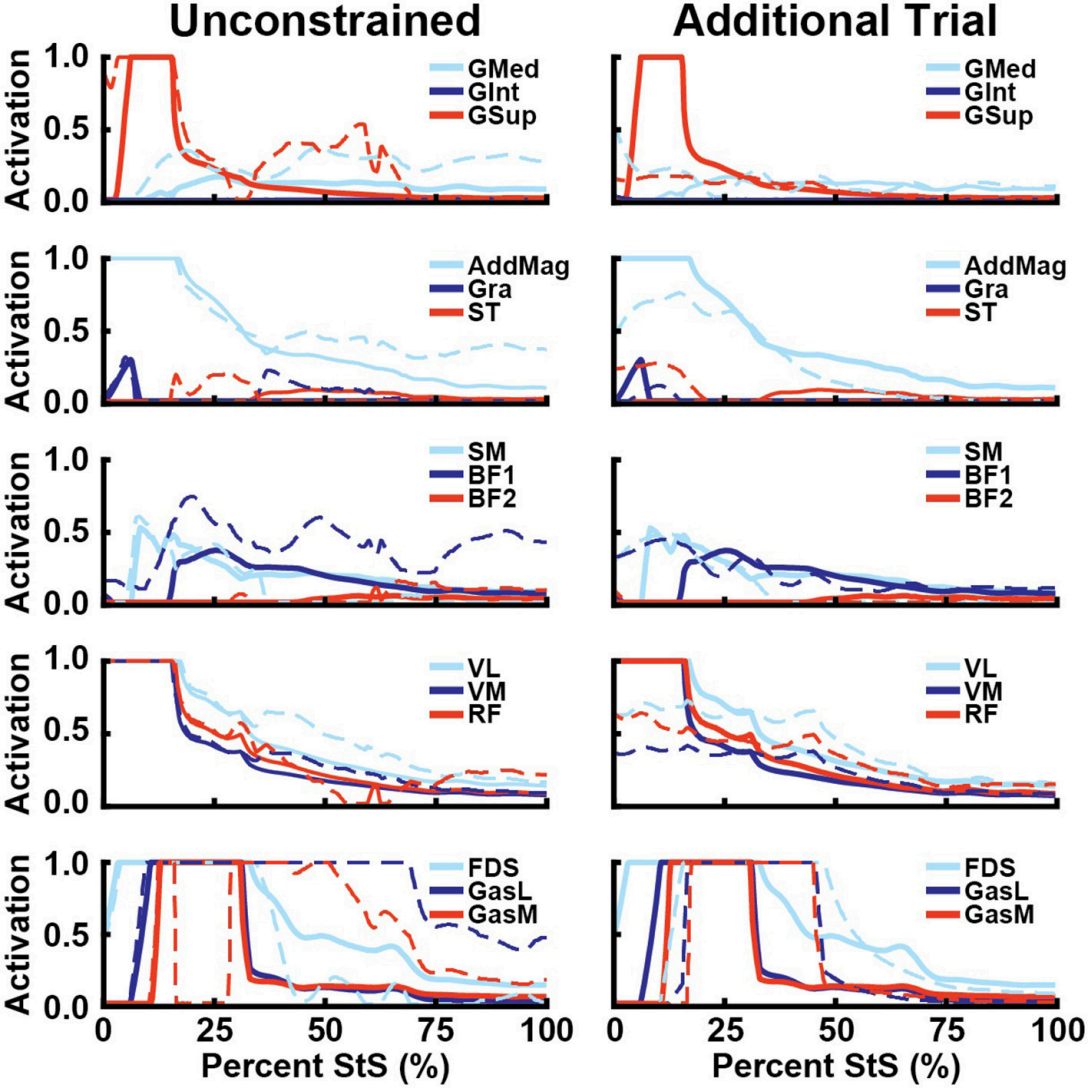
Simulated muscle activations for select hip abductors (1st row), hip extensors (2nd row), knee flexors (3rd row), knee extensors (4th row), and ankle extensors (5th row) vs. percent StS (cf. Figures 6–8). Thick lines: Nominal simulation activation. Dotted lines: Activation from alternative simulation. Unconstrained: Ankle and knee internal/external rotation and abduction and adduction followed experimental data. Additional Trial: A second complete trial was simulated using data shown in Supplementary Figures S6–S8 and Supplementary Table S1.

Including 3D knee and ankle joint rotations (Figure 9, left column; using the actual experimental data from the representative trial [Supplementary Figures S6–S8, Supplementary Table S1] rather than fixing knee and ankle rotations to zero) primarily increased add/abductor muscle activity, such as the GMed later in StS (average 143% increase, 122% peak) and BF1 (average 163% increase, 99% peak) (Figure 9). Thus, more abducted and externally rotated distal limb joints incurred greater moments about the hip that hip abductors balanced. Knee extensors were less affected in this sensitivity analysis. At the ankle, the GasL and GasM exhibited markedly prolonged maximal activation relative to the nominal simulation, with GasM having a sudden drop in activation from 1.0 to 0.0 in early StS before returning to high activation for much of the StS.

The alternative trial simulation had some changes in estimated limb muscle activations relative to the nominal simulations (Figure 9, right column). Activations were more evenly distributed for the M. gluteus group rather than GSup being the main muscle activated. Activation levels were also generally lower and more evenly distributed for other muscles including AddMag and the knee extensors, which did not reach activations of 1.0 and remained closer to 0.5 in this simulation. However, maximal activations of 1.0 were again found for the distal FDS, GasL and GasM muscles, whose activations remained high for a longer percentage of StS (Figure 9). The unusual ExtDigLong activity and force in the nominal simulation were not present when using this alternative trial or 3D distal joint rotations. Overall, while quantitative results were different in the alternative trial, the qualitative patterns were broadly similar, especially for proximal vs. distal hindlimb muscles (e.g., very high, sustained activations distally).

### Reserve actuators

Apart from a few notable exceptions, the reserve actuator values in our nominal and ± 10% TSL simulations were small relative to the inverse dynamics joint torques (< 1 Nm or ≤ 5% of average or peak inverse dynamics torques). The main exception was the ankle joint in flexion/extension, which required reserve actuator torques of >1 Nm average (~25% of the total) and >10 Nm peak (~79% of the total). Although the hip's average flexion/extension reserve actuator torque remained below 5% of the inverse dynamics value, the peak reserve torque exceeded 12%. Because hip ab/adduction muscles generated sufficient force for the movement (see section Pulmonary levels of IL-1β and CXCL1 are reduced in caspase-1/11 KO mice), hip ab/aduction reserve actuator torques remained < 1Nm or < 5% average and peak ID values throughout StS. At some instants, peak hip int/external rotation and knee flexion/extension exceeded our *a priori* thresholds of 1 Nm or 5% ID torques, but average values were below these thresholds.

Generally, increasing TSL by 10% longer tendons reduced reserve actuator torques, by as much as an order of magnitude (e.g., ankle flexion/extension reduced from ~25% to an average of 0.70% the inverse dynamics torque; Table 4), with all torques decreased to < 1 Nm or < 5% of inverse dynamics values. In contrast, 10% shorter tendons typically required greater average and peak reserve actuator torques; especially at the ankle (shifting to ~75% of average ID torques) and knee (shifting to ~100% of peak ID torques). Thus, distal limb muscles were far more sensitive to TSL alterations, and reserve actuators compensated for the reduced muscular capacity to generate the moments required for StS.

**Table 4:** Average and peak joint moments for all joints with the corresponding reserve actuator values used by each simulation.

**Inverse dynamics (ID) joint torque (Nm)**	**Average**	**Peak**	**Reserve torque (Nm): Nominal**	**Average (%ID)**	**Peak (%ID)**
Hip flexion/extension	7.10	13.56	Hip flexion/extension	0.17 (2.4)	1.67 (**12.3**)
Hip adduction/abduction	0.15	−2.69	Hip adduction/abduction	0.088 (60.5)	0.75 (28.0)
Hip internal/external rotation	0.12	2.77	Hip internal/external rotation	0.17 (149)	1.73 (**62.4**)
Knee flexion/extension	−0.41	4.80	Knee flexion/extension	0.30 (74.2)	3.07 (**63.9**)
Ankle flexion/extension	6.65	12.86	Ankle flexion/extension	1.63 (**24.5**)	10.1 (**78.6**)
**TSL+10%: reserve actuator torque**	**Average (%ID)**	**Peak (%ID)**	**TSL-10%: reserve actuator torque**	**Average (%ID)**	**Peak (%ID)**
Hip flexion/extension	0.019 (0.3)	0.30 (2.2)	Hip flexion/extension	0.29 (4.0)	2.50 (**18.4**)
Hip adduction/abduction	0.018 (12.4)	0.28 (10.5)	Hip adduction/abduction	0.073 (50.0)	0.69 (25.8)
Hip internal/external rotation	0.020 (17.5)	0.32 (11.4)	Hip internal/external rotation	0.20 (171)	1.87 (**67.6**)
Knee flexion/extension	0.031 (7.7)	0.50 (10.5)	Knee flexion/extension	0.56 (139)	4.80 (**100.0**)
Ankle flexion/extension	0.049 (0.7)	0.81 (6.3)	Ankle flexion/extension	5.00 (**75.3**)	12.81 (**99.6**)

*Extension, abduction and external rotation are represented as positive moments. Top Left: Inverse dynamics (ID) results. Top Right: Reserve actuator values used by the nominal simulation. Values in parentheses represent the value relative to the ID moment. Bottom Left and Right: Reserve actuator values for simulations with tendon slack lengths (TSL) altered by ± 10% for each muscle-tendon unit. Reserve actuator values that were both ≥5% of the ID moment and >1Nm are displayed in bold*.

## Discussion

We hypothesized for the StS transition in the cursorially adapted limbs of greyhound dogs that large joint ranges of motion would lead to large muscle length changes (close to limits of ~50% shorten/lengthening; Hypothesis 1), require moderately large (>50%) activations of antigravity (e.g., extensor) muscles, and use considerable non-sagittal joint motions enabled by large activations of hip adductor muscles (Hypothesis 2). If Hypotheses 1+2 were not rejected, then Hypothesis 3 predicted that passive tissues such as longer (or non-rigid) tendons and reserve actuator torques (non-tendinous passive contributions) would be necessary, especially in the distal hindlimbs, for achieving StS. Overall, our findings support all three of our hypotheses, as we explain below. We follow consideration of our hypotheses by synthesizing our findings for StS with the literature on limb kinematics, limb kinetics, muscle forces and activations, muscle length changes. Last, we discuss the validity, limitations and assumptions inherent to our model and simulations.

### Hypothesis 1: fibre length changes of hindlimb muscles

The AddMag (and some small hip muscles), knee extensors (VL,VM,RF) and ankle extensors (FDS, GasM, GasL) were all at or near their maximum length (~1.5 times optimal fibre length) early in StS (Figures 6–8, Supplementary Figure S5). The GasM and GasL were further from this limit than the FDS was, but were more sensitive to TSL changes (Figure 8). We had to tune our model initially (adjusting TSL values) so that all muscle fibres remained within 50% of their optimal fibre length throughout StS, or they would have been unable to produce active forces according to the Hill model (Zajac, 1989; Millard et al., 2013); and we chose to modify 6 out of 29 muscle fibres' lengths so that they would be within 20% of optimal fibre length at the end of StS. These two important modifications could bias our results and create circular logic (e.g., favouring Hypothesis 1) if they were mishandled. However, these modifications, at worst, created a bias toward muscles avoiding extremely long (or short) lengths during StS, as opposed to a bias towards more extreme lengths that would favour Hypothesis 1. Thus, we contend that our approach is appropriate for our analysis and we provisionally accept Hypothesis 1 – that muscles operate at non-optimal lengths during the movement – especially for distal hindlimb muscles.

Some muscles actively lengthened early in StS (< 25% StS: GMed, GSup, BF1) whereas others actively shortened (AddMag, Gra, SM, knee extensors) or remained essentially static (FDS, ExtDigLong, FDP; but see below). Based on these patterns and the force-length properties of muscle fibres in the Hill model used (Zajac, 1989; Millard et al., 2013), we would expect greater forces in those muscles that were actively lengthened (at long fibre lengths) vs. active at fibre lengths closer to optimal (for a given joint) – disproportionate to their activations, which we consider next.

### Hypothesis 2: activations of hindlimb muscles

Overall, our second hypothesis—that muscle activity will be high in extensor and adductor muscles during the movement—was supported. We found that several of the key hip extensors (GMed, GSup, AddMag; Figure 6), the three knee extensors (VL, VM, RF; Fig 7), and many of ankle extensors (e.g., the FDS, GasM, and GasL, Fig 8) all had activations of >0.5. The hamstring muscles (Figure 6: SM; BF1) were also fairly active, close to the 0.5 threshold. The hip adductors (mainly AddMag) were strongly active but opposed by hip abductors such as GSup. Thus, co-contraction likely exacerbated the joint torque requirements evident in Figure 5A. However, co-contraction may have been a result of AddMag and GSup being recruited to assist in producing the large hip extensor torques required early in StS; and the small hip muscles (Supplementary Figure S5) were activated to provide the external rotation torque (Figure 5A). These patterns held up reasonably well in the other simulations (Figure 9). A question then remaining is, were these activations (and forces) sufficient to satisfy the required joint moments on their own, and was this sufficiency insensitive to tendon slack lengths? We consider this under Hypothesis 3 below.

Regarding the prediction in the section above, the greatest activations (consistently ~1) and forces (>1 BW) early in StS were in the AddMag, knee extensor and FDS muscles, all of which were actively shortening from very long (sub-optimal) fibre lengths initially in StS. The latter muscles hence produced greater forces early in StS than other muscles acting around the same degrees of freedom but at fibre lengths closer to their optimal values. Force (vs. %StS) profiles for those actively over-lengthened muscles typically lagged behind activations, and increased only if the muscles were kept active for a sufficient duration to approach their optimal fibre lengths (e.g., AddMag in Figure 6; knee extensors in Figure 7; FDS in Figure 8). Contrastingly, muscles that did not change length much or were closer to optimal fibre length showed activation and force (vs. %StS) profiles that were more similar (e.g., other muscles in Figure 6).

### Hypothesis 3: contributions of hindlimb passive tissues

On the surface, our results emphasized that our greyhound model's muscles could not actuate the StS dynamics themselves, so other passive tissues were required. The large activations of muscles noted above appear to be in compensation for extreme (over-lengthened) fibre lengths (e.g., Zajac, 1989) as per Hypotheses 1 and 2. Increasing tendon slack length by 10% shifted the distal limb muscles away from this result, closer to optimal length (Figures 7, 8); but did not greatly influence the proximal, short-tendoned AddMag (Figure 6). The knee extensors were all >1.5 times optimal length if TSL was decreased by 10%. This pattern was exemplified by the RF muscle, which was extremely sensitive to tendon slack length: in the nominal simulation it began StS close to its length limit. Yet if TSL was increased by 10% the RF was active for more of StS and sustained more force early in StS (as it was closer to its optimal length for generating force). Furthermore, if TSL was decreased by 10%, the RF plunged to near-zero activation and force, and dropped below optimal length after ~25% StS. This procedure exemplifies how our rigid tendon assumption influenced the results: if the tendons would have been able to lengthen more early in StS then muscle fibres would operate over more favourable lengths, and thus should have been less active.

At the ankle, the FDS and medial and lateral gastrocnemii (GasM, GasL) are short-fibred, highly pennate muscles with long distal tendons. Original fibre lengths for these muscles in our model were < 2 cm; < 10% of TSL (Table 2). Even a small change in TSL therefore represented a substantial fraction of muscle fascicle length. To obtain reasonable (0.5 to 1.5 times optimal) muscle fibre lengths in our models, we had to lengthen the fascicles by up to ~400% and shorten TSL accordingly; Table 2, right columns). Our sensitivity analysis showed marked alterations in muscle activation values (and forces) for these muscles in particular (Figure 7; also Figure 9), attributable to the uncertainty about muscle fibre vs. tendon lengths in conventional Hill models. We expect that more complex muscle-tendon models may improve our representation of the extreme dynamics of the MTUs during StS in greyhounds, including those incorporating more detailed elastic elements within muscles, stretch-shortening effects, bulging, and other phenomena (e.g., Lichtwark and Wilson, 2005; Azizi et al., 2008; Nishikawa et al., 2012; McGowan et al., 2013). While the “black box” of tendon slack length is difficult to measure directly and incorporate empirically into models, our study reinforces its importance in simulations of large joint ranges of motion.

The sensitivity (or lack thereof) of some muscles to TSL, however, cannot completely explain the need for additional “reserve” actuators to augment those generated by the MTUs alone at some joints (i.e., muscle fibres alone, in our static simulations). When used by an optimization, these actuators may represent forces and torques exerted by soft tissues and other non-muscular supportive mechanisms (Rankin et al., 2016) rather than representing an inherent flaw in the model (see also Hicks et al., 2015). Remarkably, most degrees of freedom could, on average, be actuated throughout StS by muscles without substantial passive support (>1 Nm and ≥5% of inverse dynamics torques), although a few required assistance during peak torque demands (Table 4). Reserve actuator values were sensitive to TSL assumptions—shorter tendons placed muscle fibres on disadvantageous regions of their force-length curves, resulting in relatively larger reserve actuator torques to complete the StS dynamics. On the other hand, reserve actuator values were small in the +10% TSL simulation. Both results suggest that these reserve actuators likely are acting as a direct compensation for the non-optimal operating ranges of muscle fibres during the StS movement. Thus, tendon compliance appears to be a key component to successfully completing StS, in a potentially complex relationship with support from other passive tissues.

Our conclusion in support of Hypothesis 3 is further reinforced by the insensitivity of some key muscles to TSL: 10% longer tendons still mandated 100% activation of muscles such as GSup and AddMag (perhaps due to co-contraction around non-sagittal axes) even though the reserve actuator torques were below their limits and the muscles were generally closer to their optimal lengths. Hence, it becomes clearer how critical tendons are to these simulations; future simulations using forward dynamics with compliant tendons are certain to obtain qualitatively different findings (see also Rankin et al., 2016). It is very likely that at least the distal hindlimb tendons (and passive components of muscle) are “pre-loaded” (stretched) early in StS in greyhounds. Williams et al. (2008) noted that the FDS, in particular (and perhaps FDP), has a (external) tendon length vs. muscle fibre length ratio high enough that this MTU likely acts as a spring, and it should be (and is) very sensitive to changes to TSL (Figure 8). Although the slow, near-static motions of StS would preclude the power modulation (*sensu* Haldane et al., 2016) of more rapid motions such as jumping, pre-loading of tendons in StS should help generate forces to assist muscles that are at suboptimal lengths.

We also find cause to raise the issue of whether the 5% limit on reserve actuator torques is reasonable in the case of extreme motions such as StS. We predict that a greater proportion of the required torques early in StS would be generated by non-muscular, non-tendinous tissues, so a >5% threshold may be justifiable in movements such as StS that are not normal cyclical motions such as walking and running, for which the 5% threshold seems to have been intended (Hicks et al., 2015). A 0% limit (i.e., all torques must be muscular or tendinous) would be more speculative than this limit, in any case. Indeed, as the average and peak reserve torques with 10% longer TSL values were all < 20% of the total (inverse dynamics) torques (Table 4), one might even posit that a 20% limit is a plausible value, or that even larger torques (e.g., the large non-sagittal hip torques, 50%+ of total) could be justifiable. Nonetheless, until empirical measurements of what passive joint torques for given joint ranges of motion are available for greyhound hindlimbs (such data do not yet exist to our knowledge), there is no right or wrong answer to this conundrum.

### Limb kinematics

We found that greyhounds stand up through sequential (proximal to distal within the hindlimbs; Figure 3) extension at the hip, then knee then ankle, consistent with past examination of StS in dogs (Feeney et al., 2007) and humans (e.g., Pandy et al., 1995). There are very few quantitative data on comparable StS motions. Nickel et al. (1986) noted that mammalian carnivores tend to extend their forelimbs first whereas ruminant herbivores extend their hindlimbs first. Our greyhounds extended the hindlimbs first, for unclear reasons. Zannier-Tanner (1965) reported very diverse patterns of StS limb extension timing for mammalian herbivores, so general principles regarding these basic motions remain elusive.

Our results for maximal joint ranges of motion (RoM) also qualitatively match those for the only other StS study of dogs that we are aware of, by Feeney et al. (2007) on Labrador retrievers: they obtained mean RoMs of ~27° shoulder, 37° elbow, 70° wrist, 35° hip, 62° knee and 66° ankle vs. our values in Table 3 and Supplementary Table S1. Their RoMs tend to be greater for the forelimbs (e.g., their wrist/carpus 70° vs. our mean of only 32°; with non-overlapping standard deviations) whereas our greyhounds tended to have greater hindlimb RoMs (e.g., their ankle/tarsus 66° vs. our mean of 89°). These differences are interesting in light of Bertram's et al. (2000) and Colborne's et al. (2005) findings that, compared with Labrador retrievers, during trotting greyhounds have greater peak vertical GRFs supported by their more straightened hindlimbs (implying higher limb stiffness). StS likewise seems to involve distinct mechanisms for these two breeds; corresponding in part to differences in limb muscle architecture (Williams et al., 2008; Dries et al., 2018).

Joint RoMs during StS in greyhounds are larger for many degrees of freedom than published data indicate for locomotion in greyhounds or other canine breeds; much as Feeney et al. (2007) found for Labrador dogs during StS vs. walking (e.g., their Table 4: ~2x greater RoM for the ankle/tarsus joint). De Camp et al. (1993) (their Table 3) measured mean total RoMs in trotting greyhounds for the hip, knee and ankle at 31, 54, and 36°; less than half the RoM of the ankle joint in StS, in particular. Similarly, high accelerations in galloping greyhounds involved maximal RoM of ~35, 35, and 70° (Williams et al., 2009:their Figure 7); more closely approaching our StS ankle RoM. Indeed, the flexion/extension RoMs of greyhound limb joints in StS are much closer to their limits than in normal locomotion: e.g., in intact dog limbs, RoM is about 160° for the hip, 140° for the knee and 170° for the ankle (Newton and Nunamaker, 1985: their Tables 8–1). StS in greyhounds (Table 3) involves RoMs of 33, 56, and 52% of those maximal RoMs, so the distal limb joints in particular approach their maximal RoM in StS more than they do in locomotion.

There is some non-sagittal motion in the StS of greyhounds (Supplementary Videos S1, S3; Table 3). Interestingly however, we observed almost no unambiguous non-sagittal (< 20°) motion at the hip (or shoulder) even though such motion is possible, somewhat in contradiction to our Hypothesis 2. We had expected substantial non-sagittal motions during StS as a way to circumvent the steep evolutionary constraints on sagittal motion imposed by walking and running. Part of this contradiction may be by study design: we only examined trials where dogs began the motion from an adducted, bilaterally symmetrical posture on their bellies. This was a common behaviour used by almost all our subjects. However, many dogs chose to lie on their side while being positioned for this study, and would then frequently use non-sagittal rolling motions to stand up. These motions were much more variable, often obscured motion capture markers, and excluded from this study. Rolling during StS would involve strongly asymmetrical limb function and likely require more non-sagittal movement; bolstering that aspect of our Hypothesis 2.

Because of the large amount of uncertainty in our estimates of non-sagittal motions in our experimental data, we performed a sensitivity analysis to understand how changes in these joint angles may influence our simulations. We examined two extreme scenarios (no long-axis joint rotation vs. extreme rotation from experimental data); biologically realistic results likely lie somewhere in between. Greater long-axis rotations incurred greater muscle activity in the simulations (Figure 9). The initially selected representative trial had near-zero ankle internal rotations, which we assumed to reflect the limited rotations that are anatomically possible. However, other trials had >20–50° internal rotations (Table 3, Supplementary Table S1). We believe the latter results are artefacts of inaccurate marker placement and/or joint axis calculations (see Limitations section, below).

### Limb kinetics

Our study has provided the first dataset for limb kinetics during StS in dogs, revealing deeper biomechanical mechanisms used to accomplish the movement. We observed a rapid increase in total hindlimb vertical GRF to ~0.75 times body weight by ~25% StS, which corresponded to a decrease in total forelimb vertical GRF to ~0.25 times body weight at this same time (Figure 4, Supplementary Figure S9). As the GRFs required to complete StS are provided primarily by the hindlimbs, this reinforces our decision to simulate a hindlimb. GRFs are low during StS in greyhounds, compared with maximal speed locomotion. For example, Bryant et al. (1987) obtained forelimb and hindlimb peak vertical GRFs of >2 and ~1.5 times body weight for moderate-speed galloping. They commented that Jayes and Alexander's (1982) corresponding values of ~2.7 and 1.8 times body weight for faster galloping speeds in videos probably were underestimates. Williams et al. (2009) obtained roughly similar relative GRFs (< 1.6 times body weight) for galloping greyhounds during high acceleration.

For similar-sized subjects as ours, Williams et al. (2009) (their Figure 9) found that peak net joint torques (in extension) during high accelerations in galloping were ~40, 25, and 45 Nm for the hip, knee and ankle. These moments are, unsurprisingly, greater than peak StS joint torques: ~14, 5, and 13 Nm (Table 4). It is interesting to note that, while GRFs per limb are ~5x greater in rapid locomotion, the joint moments are only ~3-5x greater; presumably due to the low mechanical advantage (i.e., larger GRF moment arms) incurred by the crouched limb posture. Our model's key extensor muscle moment arms (for brevity, not plotted here) are generally near their lowest values early in StS and qualitatively in agreement with cadaver data (Williams et al., 2008). This reinforces that mechanical advantage is low when peak GRFs occur in StS, reflecting our Hypothesis 2 that muscle activations are high for greyhounds during StS. Such relatively high activation should apply in particular for comparisons to walking, in which peak GRFs are similar but joint moments are lower than in StS (Wentink, 1977).

We also observed a substantial craniad translation of the COP during StS in our greyhounds (Figure S4) corresponding to a shift from a plantigrade to a digitigrade foot posture (Figure 1, Supplementary Movies S1,S3) that kept joint moments lower than if the feet somehow remained in a digitigrade orientation as in standing. This pattern mirrors how humans reposition their feet and COP during StS in a “stabilization strategy” (Hughes et al., 1994). Similar patterns of low GRFs but poor mechanical advantage leading to high demands placed on limb extensor muscles prevail for humans during StS. As previously described, it is widely accepted that knee extensor moments are large in StS, which imposes strength:weight ratio limitations on individuals with muscle weakness or other deficits (Hughes et al., 1996; Janssen et al., 2002; Gilette and Hartman, 2003; Bieryla et al., 2007; Savelberg et al., 2007; Yoshioka et al., 2009). An alternative “stabilization strategy” (see below) seems to shift muscular demands from knee to hip extensors, with the benefit of greater overall mechanical advantage but balanced by an increased cost associated with the greater impulses that must be produced during StS (Van der heijden et al., 2009).

### Limb muscle forces and activations

We are not aware of *in vivo* measurements of muscle forces for dog hindlimbs, and electromyographic (EMG) data only allow general qualitative inferences about activation levels, not as % of maximal effort (0.0–1.0 range) as our simulations output. However, greyhounds and other dogs do activate the same “antigravity” muscles (e.g., GMed, GSup, BF, VL, VM, Gas, FDP, FDS; more variably, early bursts from Add, Gra, ST, SM; RF later in stance phase) to walk, trot or gallop as in StS (EMG data: Tokuriki, 1973a,b, 1974; Wentink, 1976, 1977; Goslow et al., 1981; Gregersen et al., 1998; Deban et al., 2012). Few computational models, let alone simulations, of dog hindlimbs exist but our active MTUs generally match those from a static optimization-based simulation of three-legged stance phase (Shahar and Banks-Sills, 2002).

The ExtDigLong is not typically active during stance phase in canine locomotion (Wentink, 1976), unlike our nominal simulation's estimated activation pattern that we judged questionable. Investigating this result further, we found that, due to the extremely flexed posture at the ankle, the ankle moment arm of this muscle was extensor (i.e., plantarflexor) until ~30% of StS, which explains why it was active. FDP took over its role later in StS (Figure 8) in the nominal simulation. Altered kinematics resulted in the ExtDigLong not having an ankle extensor moment arm and thus was inactive (Figure 9). If we had modelled the motions of the phalangeal joints this result might have changed further, preventing co-contraction of the ExtDigLong against the FDS and FDP.

Humans use homologous (or analogous) limb muscles to conduct StS. Studies have reported StS activity mainly for the equivalents of GSup, GMed, BF, ST, RF, VM, VL and GasM (EMG data: Roebroeck et al., 1994; Pandy et al., 1995; Khemlani et al., 1999; Actis et al., 2018) in addition to M. tibialis anterior and M. soleus (e.g., Silva et al., 2013; these muscles are not active/present in our simulation) e.g.,. Increased activity occurs if the former muscles if load is added to subjects, whereas other muscles (M. soleus, M. tibialis anterior) do not increase activity. Savelberg et al. (2007) explained this phenomenon as evidence for a decreased strength:weight ratio in loaded subjects, challenged by StS biomechanical demands that the primary StS muscles satisfied. Furthermore, StS in humans tends to involve marked co-activation of muscles with antagonist (e.g., hip/knee flexor) actions against antigravity muscles, perhaps as to aid in stability (e.g., Roebroeck et al., 1994; Khemlani et al., 1999; Savelberg et al., 2007; de Souza et al., 2011; Actis et al., 2018; Shia et al., 2018).

The common muscle coordination strategy in StS found in humans favours key antigravity muscles along with some coactivation of antagonist muscles and is similarly observed in our dog simulations, Our study therefore hints at a broader pattern that might prevail across mammals or even tetrapods—a subject worthy of further inquiry. We predict, though, that there should be major differences in StS for some species such as quadrupeds, in which a “stabilization strategy” involving increased trunk pitch early in StS (e.g., Savelberg et al., 2007; Van der heijden et al., 2009) e.g., might not be feasible as an alternative StS strategy. Thus proximal-to-distal “momentum transfer” strategies within the limbs might be more commonplace outside of bipeds, in addition to varied function of fore- vs. hindlimbs (as in labradors vs. greyhounds; above). Oddly however, our simulations did not estimate an activation sequence of muscles from proximal to distal: key antigravity muscles all became maximally active immediately with StS initiation (Figures 6–8), which is unlike in human StS and unlike our proximal-distal joint kinematics pattern, but could be an artefact of our static optimization criteria (Pandy et al., 1995; also see below). Regardless, distal limb muscle activations seemed to remain high for longer during StS relative to proximal limb muscles, and this was relatively insensitive to the input parameters we varied.

### Limb muscle length changes

While *in vivo* or modelling data on hindlimb muscle length changes are scarce for locomotor behaviours in greyhounds or other dog breeds (or other species), prevailing evidence indicates more isometric patterns for most limb muscles, keeping muscles closer to their optima for force production (e.g., Cutts, 1989; Roberts et al., 1997; Burkholder and Lieber, 2001; Maganaris, 2001; Rubenson et al., 2012). However, Goslow et al. (1981) used motion analysis, EMG data and a simple 2D model of dog limbs to infer MTU length changes during a variety of gaits up to 6.7 ms^−1^, suggesting that GMed and BF actively shortened during stance phase, whereas VL and GasM actively lengthened then shortened in stance (i.e., MTUs acting like springs). Contrastingly, GMed and BF actively lengthened whereas VL actively shortened and GasM actively lengthened then shortened (both to modest amounts) during StS in our greyhound simulations (Figures 6–8)– indicating clear differences in MTU work and power for locomotion vs. StS that future studies should pursue.

Our actively shortening SM and actively lengthening VL in StS, however, qualitatively match direct sonomicrometry measurements in jumping and running dogs, as follows. The SM fascicles exhibited a < 30% change from resting length with a ~120° knee extension RoM in jumping (Gregersen and Carrier, 2004). The VL fascicles had a < 7% length change (or >20% if full period of EMG activity used) during stance phase (RoMs not reported) in running (Gregersen et al., 1998). Thus, at least for muscle fascicle length changes in two demanding behaviours and two muscles, MTU length changes in StS grossly match (cf. latter studies vs. our Figures 6, 7). StS in humans and dogs shows some interesting similarities and differences in terms of MTU or fascicle length changes. Using experiments and a simple 2D model similar to Goslow's et al. (1981), Roebroeck et al. (1994) estimated that there was active shortening in the human equivalents of GSup, BF, ST, VL and VM early in StS (followed by lengthening), whereas the RF and distal limb muscles were isometric or barely actively lengthening. These, especially VL and VM (Figure 7), roughly correspond to our patterns of active muscle length change except for the GSup and BF1 which actively lengthened (Figure 6); our GasL and GasM results (modest active stretch-shortening) are more ambiguous comparisons (Figure 8).

While tendons surely play an important role in StS (see below), we predict little potential for elastic energy storage; unlike in locomotion or jumping (e.g., Gregersen et al., 1998; Gregersen and Carrier, 2004). This is because StS involves relatively slow (quasi-static), vertical motions (without a quick countermovement-style stretch) that should disfavour rapid conversion of elastic strain energy into kinetic energy to raise the body's centre of mass (or move it forwards). Similarly, Hughes et al. (1996) found that static and dynamic methods produced similar results for StS in humans, and Pandy's et al. (1995) simulations favoured non-ballistic optimization criteria.

### Validation, limitations and assumptions

Our experimental data involved kinematic data from multiple markers placed on each segment to estimate 3D joint motions in StS from our greyhound subjects. Small differences in marker placement have been observed to substantially alter measured joint angles (Kadaba et al., 1990). Combined with the substantial individual variability in StS behaviour that was evident within and among individuals, this helps to explain the variation in our kinematic data (e.g., Table 3, Supplementary Table S1). Improved 3D kinematic data would augment our results by reducing uncertainties regarding the limb joint motions used in the simulations. However, increasing the number of markers was prohibitive because of prolonged experimental setup time, and could alter the StS motion by creating discomfort or obstructing joint mobility. Regardless, the simplification we adopted was an appreciable step forward from the only other published study of StS in non-humans that we are aware of, involving 2D kinematics in Labrador retriever dogs (Feeney et al., 2007).

As Table 2's “Fmax, Williams et al. (2008)” column indicates, our dissected individual had muscles that were on average about 50% weaker in maximal isometric force generation capacity than an average racing greyhound, except in the case of M. gluteus medius and M. tensor fascia lata which were ~11 and 25% stronger. Additionally, our model incorporated six muscles that were not in the Williams et al. (2008) dataset; and Williams's et al. (2009: Supplementary Table A1) subjects were estimated to have had more massive hindlimbs at comparable body masses (see Methods above). While subjective (investigator) and measurement errors may have contributed, a large part of this difference may come from choice of subjects: we studied normal, domesticated, household greyhounds at a range of ages and fitness rather than using active athletes as were those studied by Williams et al. (2008, 2009). A simulation of a more active athlete thus should have lower muscle activations due to larger muscle areas; if so, our Hypothesis 1 regarding length changes might remain unaffected but Hypotheses 2 and 3 (muscle activations and passive tissue support) might be weakened. Regardless, for our study's intent to focus on “normal” greyhound pet subjects, we contend that our anatomical model is sufficient.

By using an extensive experimental dataset for StS and anatomically realistic model, we simulated muscular mechanics in a greyhound in unprecedented detail while maximizing the rigor of the data involved. Tests of the validity of our model and simulations are less of a concern under these conditions, except in key areas as follows. Our assumption that tendons were rigid was intentionally unrealistic, allowing us to tease apart how muscles alone may contribute to StS. It is interesting that muscles can successfully drive StS in our greyhound simulation; with some quantifiable passive support at more joints than others. We addressed this assumption of rigid tendons in more detail with our sensitivity analysis of tendon slack length and reserve actuator torques. Further sensitivity analyses of the input experimental data strengthened our hypothesis testing. However, some concerns remain. Certainly, the “gold standards” of validation (see Hicks et al., 2015; Rankin et al., 2016) including electromyographic data on muscle activity, sonomicrometry or ultrasound measurements of muscle/tendon length change, and biplanar fluoroscopic measurements of 3D joint motions during StS would improve future applications of this simulation approach and test its accuracy.

Tendon slack length (TSL) values are a related limitation. We “tuned” our TSL values to keep muscle fibre lengths in our model within a reasonable operating range across a wide RoM. This, however, might lead our model to be less able to simulate non-StS motions such as walking or running without re-tuning TSL values. There is no agreement in the literature on what kind of tuning is “correct” for the black box of TSL (e.g., Scovil and Ronsky, 2006; Redl et al., 2007—yet see Dries et al., 2016, 2018), so this is an issue that future implementations of the model should confront. Furthermore, experimental data (e.g., Roberts et al., 1997; Herbert et al., 2002) suggest that conventional assumptions about muscle vs. tendon length changes may be overly simplistic.

We used static optimization to generate our simulations, which included an objective function that minimized the sum of muscle activations squared (as did Actis et al., 2018; for human StS). However, this approach likely generates results that do not exactly match the controls that greyhounds actually use in StS and may explain many disparities in our results vs. other data. Pandy et al. (1995) conducted a sophisticated forward dynamic simulation of StS in humans, finding that the time-derivative of muscle force seemed to work best as the optimization criterion. Indeed, we expect that their algorithm would give better results (i.e., more gradual activation of limb muscles, perhaps in starker proximal-distal sequence) for greyhound StS. Later, Bobbert et al. (2016) challenged that minimizing the sum of squared “control effort” (equivalent to activation from 0 to 1 in our simulations) might be a better choice. Resolution of this issue awaits more study of what different species optimize in StS decisions (see also Erdemir et al., 2007).

## Conclusions

Our models and simulations have considerable uncertainties and assumptions, yet even in light of these we contend that our combined experimental and computational analysis of StS dynamics in greyhounds supports our hypotheses that key antigravity hindlimb muscles operate close to their limits of length change, and even perhaps force (and thus activation and mechanical work) during StS. We infer that this proximity to biomechanical limits requires substantial contributions from soft tissues including tendons and perhaps ligaments or other arthrological features in order to achieve StS.

Although the limb forces in StS are less than in high-speed locomotion and more comparable to the forces experienced during walking, the unfavourable mechanical advantage of the limb joints requires substantial forces and activations from the limb muscles. This requirement is only amplified by the increased length changes of the muscles required to produce the measured joint ranges of motion, moving the animals from a crouched, supine position to an erect, upright limb orientation and using most of the feasible ranges of motion of the joints themselves as well as the muscles. Relative to normal walking and running, non-sagittal motions are increased in the StS behaviour we focused on here and do impose some extra demands on muscles such as hip adductors. Alternative StS strategies for quadrupeds include rolling behaviours that should exaggerate non-sagittal motions in return for poorly understood mechanical benefits, and limb coordination patterns that should involve greater force production and length changes from the limb muscles.

Our study has shown how non-locomotor biomechanical demands deserve further consideration in the study of how the musculoskeletal system is adapted to, and constrained by, these demands vs. the more conventional research focus on walking and running. We have elucidated how greyhounds can sustain some aspects of the sit-to-stand transition with their muscles but need passive tissues, including but not necessarily limited to tendons, to fully achieve it because of their cursorial limb structure's emphasis on short-fibred distal muscles and adaptation to parasagittal, upright-limbed locomotion.

## Author contributions

All authors contributed to the writing of the manuscript and gave final approval for publication. RE helped design the experiments and simulations and analysed the data. JR helped develop software, run simulations and assisted with data interpretation. JH conceived and guided the project and contributed to the experimental design and simulations and assisted with data interpretation.

### Conflict of interest statement

The authors declare that the research was conducted in the absence of any commercial or financial relationships that could be construed as a potential conflict of interest.
