# Supplementary material for: Limb Kinematics, Kinetics and Muscle Dynamics During the Sit-to-Stand Transition in Greyhounds
Source: Front Bioeng Biotechnol. 2018 Nov 16;6:162. doi: 10.3389/fbioe.2018.00162 (PMC6250835; doi:10.3389/fbioe.2018.00162)
Supplement: Supplementary file 1 [file Table_1.DOCX]

**Supplementary Table S1:**

Mean and standard deviation (SD) of the range of motion for each greyhound forelimb joint in the nominal trial (top) and for all fore- and hindlimb joints in the additional trial used to test simulation sensitivity (bottom). Data were obtained calculated from all trials (21 trials from 4 dogs for forelimb, 39 trials from 6 dogs for hindlimb). The “Trial” columns indicate the values for the specific trials used for simulations (compare with Table 3; also Figs. S6-S8). Values lying outside 1 SD from the mean data are emphasized in bold font.

|  | Flexion/Extension | | | | | | | Add/Abduction | | | | | | | | Internal/External Rotation | | | | | | | |  |
| --- | --- | --- | --- | --- | --- | --- | --- | --- | --- | --- | --- | --- | --- | --- | --- | --- | --- | --- | --- | --- | --- | --- | --- | --- |
| Joint | Mean | | ± | | 1 S.D. | | Trial | Mean | | | ± | 1 S.D. | | | Trial | Mean | | ± | | 1 S.D. | | | Trial |  |
| FORELIMB |  |  | |  | |  | | |  |  | | |  |  | | |  | |  | |  |  | | |
| Shoulder | 20.0 | ± | | 6.0 | | **11.7** | | | 9.2 | ± | | | 3.1 | **14.1** | | | 12.2 | | ± | | 7.7 | 16.1 | | |
| Elbow | 53.4 | ± | | 25.3 | | 39.8 | | | 11.3 | ± | | | 4.5 | 15.2 | | | 18.3 | | ± | | 6.0 | 21.6 | | |
| Wrist | 32.2 | ± | | 12.7 | | 31.8 | | | 8.9 | ± | | | 4.1 | **13.8** | | | 17.0 | | ± | | 12.3 | **35.4** | | |
| OTHER TRIAL | | | | | | | | | | | | | | | | | | | | | | | | |
| Shoulder | 20.0 | ± | | 6.0 | | **29.9** | | | 9.2 | ± | | | 3.1 | 10.3 | | | 12.2 | | ± | | 7.7 | **30.2** | | |
| Elbow | 53.4 | ± | | 25.3 | | 52.4 | | | 11.3 | ± | | | 4.5 | 11.3 | | | 18.3 | | ± | | 6.0 | **32.4** | | |
| Wrist | 32.2 | ± | | 12.7 | | 32.5 | | | 8.9 | ± | | | 4.1 | **23.9** | | | 17.0 | | ± | | 12.3 | **43.0** | | |
| Hip | 53.4 | ± | | 5.6 | | **40.4** | | | 18.5 | ± | | | 9.9 | 9.6 | | | 8.7 | | ± | | 4.3 | **13.4** | | |
| Knee | 78.5 | ± | | 10.7 | | **40.5** | | | 30.2 | ± | | | 10.2 | **13.9** | | | 25.7 | | ± | | 4.9 | **8.7** | | |
| Ankle | 88.7 | ± | | 15.7 | | **64.2** | | | 23.5 | ± | | | 13.3 | 14.4 | | | 51.3 | | ± | | 13.7 | **21.8** | | |
