# Supplementary material for: Limb Kinematics, Kinetics and Muscle Dynamics During the Sit-to-Stand Transition in Greyhounds
Source: Front Bioeng Biotechnol. 2018 Nov 16;6:162. doi: 10.3389/fbioe.2018.00162 (PMC6250835; doi:10.3389/fbioe.2018.00162)
Supplement: Supplementary file 5 [file Data_Sheet_1.PDF]

Limb kinematics, kinetics and muscle dynamics during the sit-to-stand transition in greyhounds

Richard G. Ellis[1], Jeffery W. Rankin[1,2], and John R. Hutchinson[1]*
[1]Structure & Motion Lab, The Royal Veterinary College, North Mymms, Hertfordshire, United Kingdom.
[2]Pathokinesiology Laboratory, Rancho Los Amigos National Rehabilitation Center, Downey, CA 90242, USA.

*Correspondence:
Prof. John R. Hutchinson
jhutchinson@rvc.ac.uk

Supplementary Text S1:
Experimental Data Inclusion Criteria, Coordinate System Methods, Scaling of Model, Results: Datasets 1 vs. 2, and Results: Forelimb Biomechanics in StS

Experimental Data Inclusion Criteria

Our initial experimental dataset ("dataset 1" and "dataset 2" combined) was 129 trials from 8 dogs, for the forelimbs and hindlimbs. To reduce this original dataset down to the trials used for analysis, we first applied a set of initial inclusion criteria: (1) dog started StS from a belly-lying prone position; (2) dog completed StS successfully; (3) dog did not adjust left fore/hind foot placements; (4) dog had no forward motion (e.g. walking) during StS; (5) StS was bilaterally symmetrical. This led to 73 forelimb trials from 4 dogs and 84 hindlimb trials from 2 dogs being excluded; leaving 56 forelimb trials from 4 dogs and 45 hindlimb trials from 6 dogs.

Next, we required any trials to be considered further to have sufficient motion capture data so that 3D limb kinematics could be calculated throughout a full StS cycle. This excluded 22 forelimb trials and 4 hindlimb trials but led to no dogs being entirely excluded from the study. Furthermore, we required all four limbs to be on force plates, which excluded another 2 forelimb and 2 hindlimb trials, but no dog subjects.

Finally, we removed individual joint data that were judged as outliers (~45° from group mean); excluding 11 forelimb trials but no hindlimb trials, and no dogs. This produced our final dataset of 21 forelimb trials from 4 dogs and 39 hindlimb trials from 6 dogs.

Coordinate System Methods

We used three markers on each segment to define right-handed technical coordinate systems. For all joints, the x-axis was cranio-caudal (positive is cranial), the y-axis was medio-lateral (positive is to the dog's left) and the z-axis was dorso-ventral (positive is dorsal). As we introduced additional anatomical markers over the course of data collection (see Methods; here termed "Datasets 1 vs. 2"), the points used to define our axes varied between the first four subjects and the last four. The body axes definitions were the same for both data subsets. All coordinate systems presented here are for the left leg.

Body:

z-axis: cross product of the unit vector pointing from the left pelvis (body 5) to the right pelvis marker (body6) with the x-axis.

y-axis: cross of the z- and x-axes.

x-axis: unit vector pointing from the left pelvis marker (body 5) to the left back marker (body 3) from each body plate.

<u>First four subjects (Dataset 1):</u>

Humerus (Upper Arm):
- z-axis: unit vector from ElbL to Should.
- y-axis: cross product of the z-axis with the unit vector from Should to UA2.
- x-axis: cross product of the y- and z-axes.

Radius/Ulna (Lower Arm):
- z-axis: unit vector from WristL to ElbL.
- y-axis: cross product of the z-axis with the unit vector from WristL to LA2.
- x-axis: cross product of the y- and z-axes.

Forefoot:
- z-axis: unit vector from MCHL to WristL.
- y-axis: cross product of the z-axis with the unit vector from MCHL to Forefoot1.
- x-axis: cross product of the y-and z-axes.

Femur (Thigh):
- z-axis: unit vector from KneeL to Hip.
- y-axis: cross product of the z-axis with the unit vector from KneeL to Thigh1 .
- x-axis: cross product of the y- and z-axes.

Tibia/Fibula (Shank):
- z-axis: unit vector from AnkleL to KneeL.
- y-axis: cross product of the z-axis with the unit vector from AnkleL to Shank2 .
- x-axis: cross product of the y- and z-axes.

Hindfoot:
- z-axis: unit vector from MTHL to AnkL.
- y-axis: cross product of the z-axis with the unit vector from MTHL to Hindfoot1.
- x-axis: cross product of the y-and z-axes.

<u>Last four subjects (Dataset 2):</u>

Humerus (Upper Arm):
- z-axis: unit vector from ElbL to Should.
- y-axis: cross product of the z- and x-axes
- x-axis: cross product of the z-axis with the unit vector from ElbL to ElbM.

Radius/Ulna (Lower Arm)
- z-axis: unit vector from WristL to ElbL.
- y-axis: cross product of the z- and x-axes.
- x-axis: cross product of the z-axis with the unit vector from WristL to WristM.

Forefoot:
- z-axis: unit vector from MCHL to WristL.
- y-axis: cross product of the z-and x-axes.
- x-axis: cross product of the z-axis with the unit vector from WristL to WristM.

Femur (Thigh):
- z-axis: unit vector from KneeL to Hip.
- y-axis: cross product of the z- and x-axes.
- x-axis: cross product of the z-axis with the unit vector from KneeL to KneeM.

Tibia/Fibula (Shank):
- z-axis: unit vector from AnkleL to KneeL.

y-axis: cross product of the z- and x-axes.
x-axis: cross product of the z-axis with the unit vector from KneeL to KneeM.

Hindfoot
z-axis: unit vector from MTHL to AnkL.
y-axis: cross product of the z-and x-axes.
x-axis: cross product of the z-axis with the unit vector from AnkL to AnkM.

Scaling of Model to Fit Experimental Subject

As per the Methods, the model was scaled in Opensim to match the subject's dimensions, and the scaling factors of model to experimental subject size were:
Pelvis segment: 0.81 * Model animal size
Femur segment: 0.89 * Model animal size
Tibia segment:0.76 * Model animal size
Foot segment:0.78 * Model animal size

Results: Datasets 1 vs. 2

Datasets 1 and 2 showed generally similar patterns of hindlimb joint flexion/extension but differed noticeably in some non-sagittal motions, as Supplemental Figure S10 shows. In particular, adduction of the hip, knee and ankle and internal rotation of the knee (early in StS) and ankle (late in StS) showed the most divergence between the two datasets. Furthermore, variability of the kinematic data from dataset 1 was often greater, such as for hip flexion/extension and almost all of the non-sagittal motions.

Together, these results are consistent with the inference that the greater number of motion capture markers in dataset 2 helped produce more consistent and thus probably more reliable data on 3D hindlimb joint motions in our greyhound subjects, especially in ankle internal/external rotation and add/abduction. This is reassuring as the subject used for both our representative and additional experimental trials for simulations was our fifth subject in both cases, and thus from dataset 2. Thus the quality of experimental data used in our simulations should be adequate for the purpose of broadly outlining the kinematics during StS; a conclusion bolstered by the reasonable fit of those trials to the mean kinematic and kinetic datasets (Figs. 3,4,S2-S4,S6-S8-S10; Tables 3,S1).

Results: Forelimb Biomechanics in StS

In the forelimbs of our greyhounds (Fig. S9A,C,E) there was minimal shoulder joint extension during StS. At the elbow, we observed slow extension throughout StS. The wrist dorsiflexed up to about 50% of the StS cycle with less joint motion thereafter. The maximal forelimb joints' ranges of extension were ~20-53°; largest at the elbow, but motions overall were smaller than in the hindlimbs (Table S1). Scapular rotations could not be quantified due to skin motion.

Upward acceleration generated by the hindlimbs was somewhat balanced by reduced forelimb forces (Fig. S9B,D,F) before ~40% of StS, although these vertical GRFs from the forelimbs gradually increased through the remainder of StS. Caudad acceleration from the forelimbs began early in StS, declined to near-zero around 20% of StS, and then increased again and tended to reach a plateau by 40-50% StS, resulting in nearly even weight distribution between the fore- and hindlimbs during stance. Fig. S4 shows that the forelimbs' centre of pressure (CoP) was more variable than the hindlimbs, owing to much more varied placements of the forelimbs; however, the representative (and additional) trial used in simulations had a CoP that was near the midline.
