# Supplementary material for: Limb Kinematics, Kinetics and Muscle Dynamics During the Sit-to-Stand Transition in Greyhounds
Source: Front Bioeng Biotechnol. 2018 Nov 16;6:162. doi: 10.3389/fbioe.2018.00162 (PMC6250835; doi:10.3389/fbioe.2018.00162)
Supplement: Supplementary file 6 [file Data_Sheet_2.PDF]

Limb kinematics, kinetics and muscle dynamics during the sit-to-stand transition in greyhounds

Richard G. Ellis[1], Jeffery W. Rankin[1,2], and John R. Hutchinson[1]*
[1]Structure & Motion Lab, The Royal Veterinary College, North Mymms, Hertfordshire, United Kingdom.
[2]Pathokinesiology Laboratory, Rancho Los Amigos National Rehabilitation Center, Downey, CA 90242, USA.

*Correspondence:
Prof. John R. Hutchinson
jhutchinson@rvc.ac.uk

SUPPLEMENTARY ONLINE INFORMATION

Supplementary Figure S1:
Body mass and leg length regression analysis. Because we used a disembodied greyhound pelvis with intact hindlimbs to create the musculoskeletal model, torso length and body mass were estimated. To calculate body mass and moments of inertia we performed an ordinary least-squares linear regression of hind limb length against body mass and body length for the eight experimental dogs. We used this to predict body mass and length of the model animal. We found that body mass=0.787*leg length-14.845 ($R^2 = 0.794$), and torso length=0.895*leg length+13.612 ($R^2 = 0.558$). Reduced major axis ("model II") regression was deemed unnecessary because body masses (x values) were known.

Supplementary Figure S2: Int/External Rotation Kinematics
Internal (-) and external (+) rotations for the shoulder (A), hip (B), elbow (C), knee (D), wrist (E) and ankle (F) versus percent StS. Solid black line: group average kinematics, averaged first within a dog and then across dogs. Dotted black line: ±1 standard deviation. Solid red line: Joint trajectory of the representative trial used for simulation.

Supplementary Figure S3: Add/Abduction Kinematics
Adduction (-) and abduction (+) angles for the shoulder (A), hip (B), elbow (C), knee (D), wrist (E) and ankle (F) versus percent StS. Solid black line: group average kinematics, averaged first within a dog and then across dogs. Dotted black line: ±1 standard deviation. Solid red line: Joint trajectory of the representative trial used for simulation.

Supplementary Figure S4: Centre of Pressure (CoP) Calculations
Force plate measurements of the centre of pressure (CoP) for the hindlimbs (on one plate) and forelimbs (on a second plate) for greyhounds during StS, plotted as anteroposterior (x-axis) versus mediolateral (y-axis) coordinates. Data were normalized to hindlimb leg length. Black Lines: individual trial data. Red Line: Nominal trial used for hindlimb simulation. Forelimb CoP and forces were not used in any simulations. The total hindlimb CoP (middle of three lines) was broken into individual limb CoPs (left and right lines) and GRFs as per the Methods. CoP traces

(for both fore- and hindlimbs) started caudally at the beginning (~0%) of StS and moved cranially throughout StS.

Supplementary Figure S5: Simulation Results for Small Hip Muscles
Simulated muscle activation, body weight-normalized muscle force ("xBW"), and normalized fibre length for small hip muscles versus StS. Only muscles where activation exceeded 20% of maximum are shown. Solid line: Nominal simulation. Dotted and Dashed lines: simulation results using altered tendon slack lengths (±10% of nominal).

Supplemental Figure S6: Additional Trial Flexion/Extension Kinematics
Corresponds to Figure 3, using kinematic data for the other trial, shown for validation of our trial choice. Flexion (-) and extension (+) angles for the shoulder (A), hip (B), elbow (C), knee (D), wrist (E) and ankle (F) joints versus percent StS cycle (see Methods). Solid black line: group average kinematics, averaged first within a dog and then across dogs. Dotted black lines: ±1 standard deviation. Solid red line: Joint trajectory of the additional trial chosen for simulation.

Supplementary Figure S7: Additional Trial Int/External Rotation Kinematics
Corresponds to Figure S2. Internal (-) and external (+) rotations for the shoulder (A), hip (B), elbow (C), knee (D), wrist (E) and ankle (F) versus percent StS. Solid black line: group average kinematics, averaged first within a dog and then across dogs. Dotted black line: ±1 standard deviation. Solid red line: Joint trajectory of the additional trial chosen for simulation.

Supplementary Figure S8: Additional Trial Add/Abduction Kinematics
Corresponds to Figure S3. Adduction (-) and abduction (+) angles for the shoulder (A), hip (B), elbow (C), knee (D), wrist (E) and ankle (F) versus percent StS. Solid black line: group average kinematics, averaged first within a dog and then across dogs. Dotted black line: ±1 standard deviation. Solid red line: Joint trajectory of the additional trial chosen for simulation.

Supplementary Figure S9: Experimental Forelimb Kinematics and Kinetics
Experimental flexion (-) and extension (+) angles for the shoulder (A), elbow (C) and wrist (E) joints and measured forelimb ground reaction forces (normalized by body weight; BW^{-1}) in the craniocaudal (B), mediolateral (D) and vertical/dorsoventral (F) directions versus percent StS cycle (see Methods). Solid black line: group average kinematics/kinetics, averaged first within a dog and then across dogs. Dotted black lines: ±1 standard deviation. Solid red line: Kinematics/kinetics for the representative trial chosen for forelimb simulation (see also Supplementary Table S1). Supplementary Figures S2,S3 show all data for non-sagittal joint trajectories.

Supplementary Figure S10: Experimental Marker Datasets 1 vs. 2
As per the Methods, we used different marker sets for some subjects early/late in the study. This figure shows the overall kinematic patterns produced by the initial (Dataset 1) and later (Dataset 2) motion capture data from the greyhound subjects' hindlimbs. See Supplementary Text S1 for further discussion.

Supplementary Video S1: Video of Dog Standing Up
Example trial.

Supplementary Video S2: Segmented Images of Thigh Muscles of Left Greyhound Hindlimb

Showing muscles in colour as follows (abbreviations are in Table 2), in order as visible from cranial to caudal on first and middle frames: green: Sart, yellow: TFL (2 parts), yellow-green: GMed, magenta: VL, dark pink: BF (both parts), light purple (near GMed): GSup, red: ST, pinkish-brown: SM, turquoise: Gra, green (near Gra): AddMag, pink: AddBrev, blue: VM, pinkish-brown (almost parallel to Sart in green): Sart2, purple: IP, light blue: RF.

Supplementary Video 3: Simulation of Dog Standing Up

Showing the nominal representative trial, including GRF and CoP kinetic data (arrow), in oblique left lateral view of the hindlimb, slowed down 10 times. Yellow spheres on the body and limbs show marker locations as per Figure 1.
