# Supplementary figures and images for: Limb Kinematics, Kinetics and Muscle Dynamics During the Sit-to-Stand Transition in Greyhounds
Source: Front Bioeng Biotechnol. 2018 Nov 16;6:162. doi: 10.3389/fbioe.2018.00162 (PMC6250835; doi:10.3389/fbioe.2018.00162)

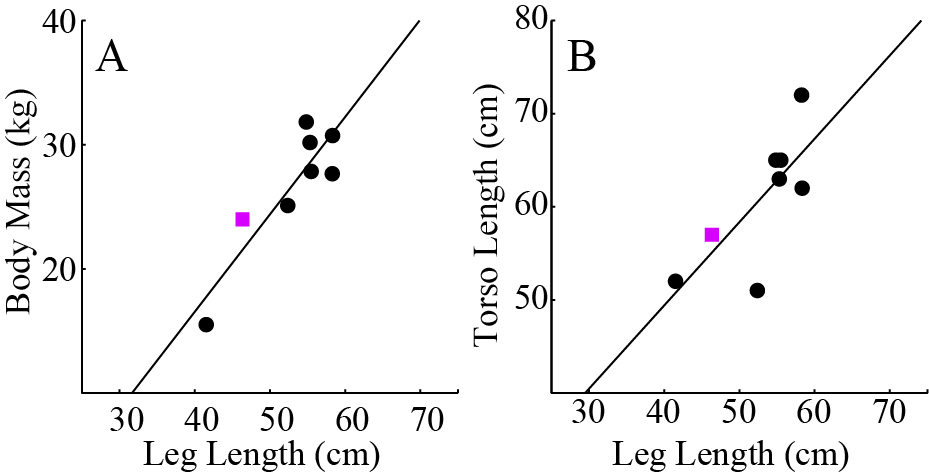

Supplement: Supplementary file 7 [file Image_1.JPEG]

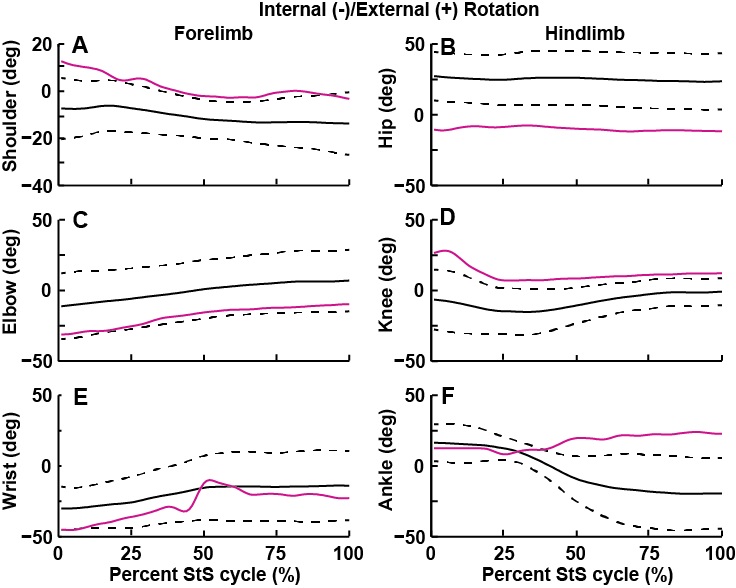

Supplement: Supplementary file 8 [file Image_2.JPEG]

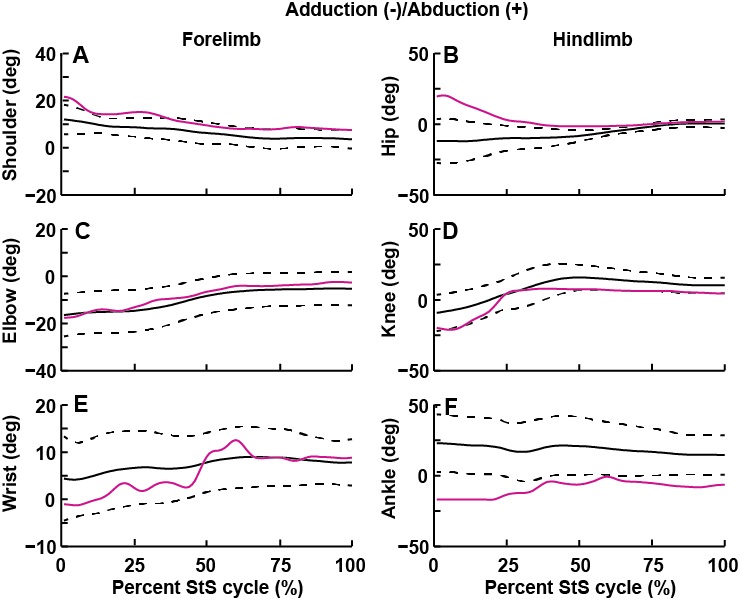

Supplement: Supplementary file 9 [file Image_3.JPEG]

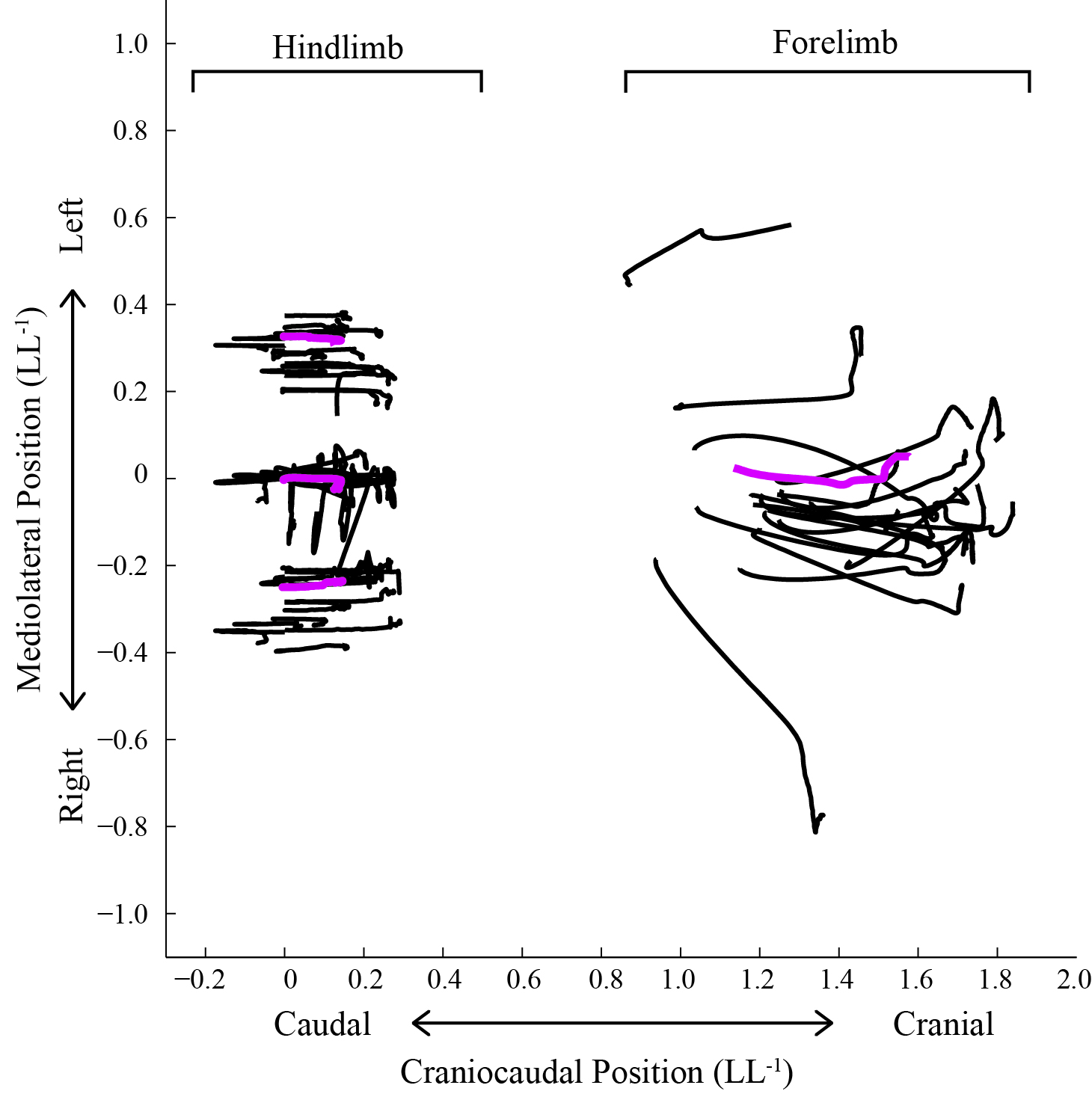

Supplement: Supplementary file 10 [file Image_4.JPEG]

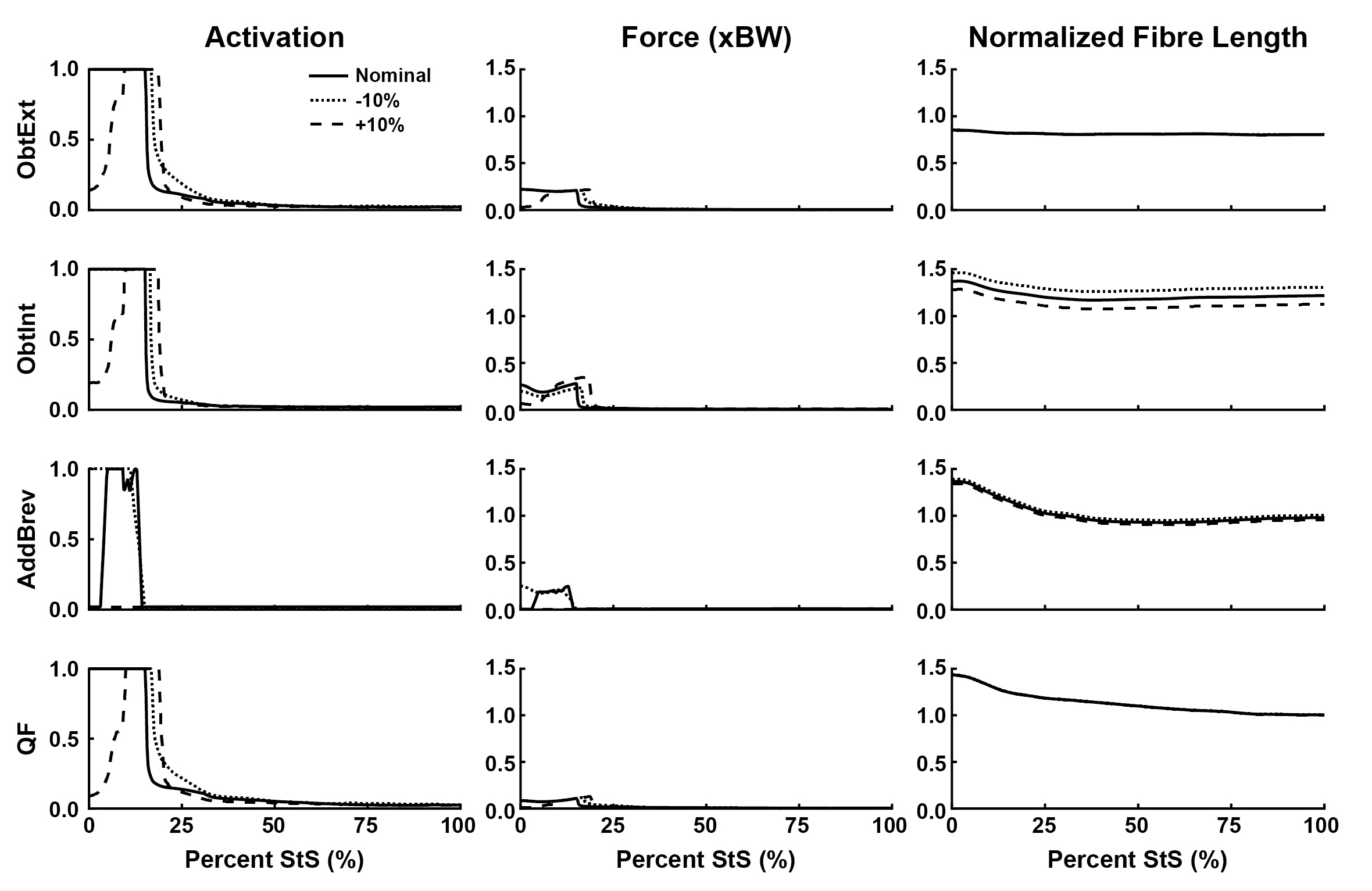

Supplement: Supplementary file 11 [file Image_5.JPEG]

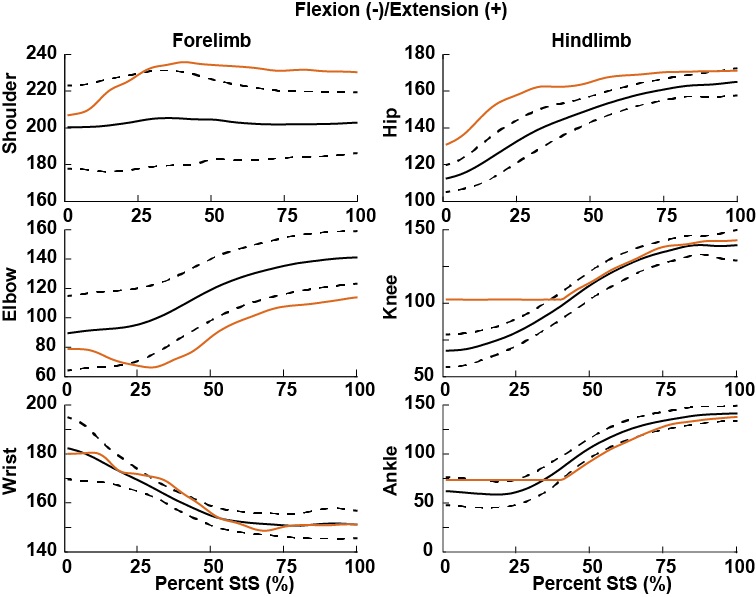

Supplement: Supplementary file 12 [file Image_6.JPEG]

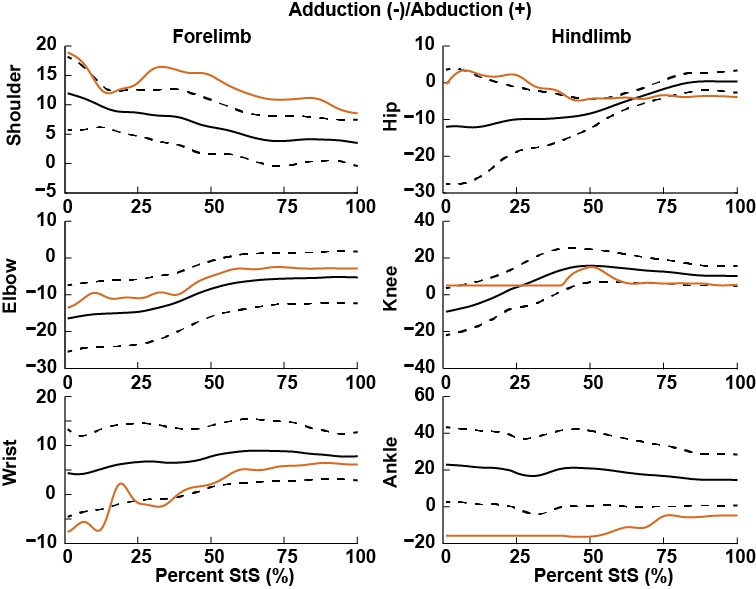

Supplement: Supplementary file 13 [file Image_7.JPEG]

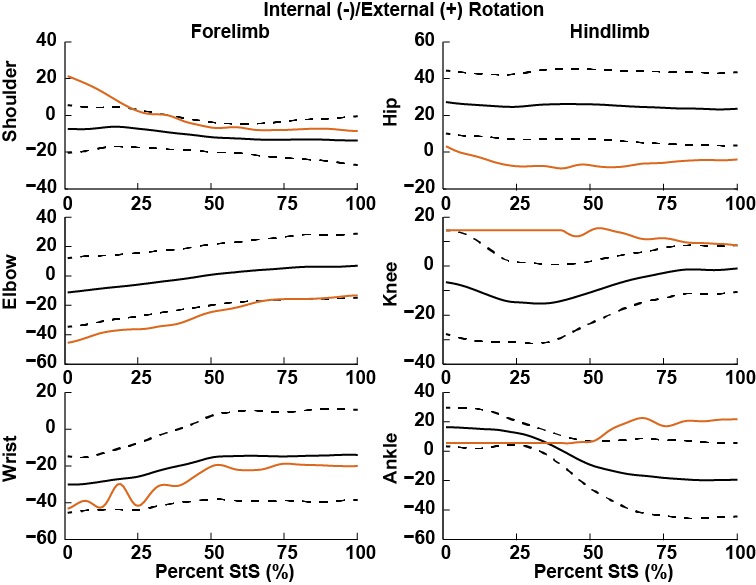

Supplement: Supplementary file 14 [file Image_8.JPEG]

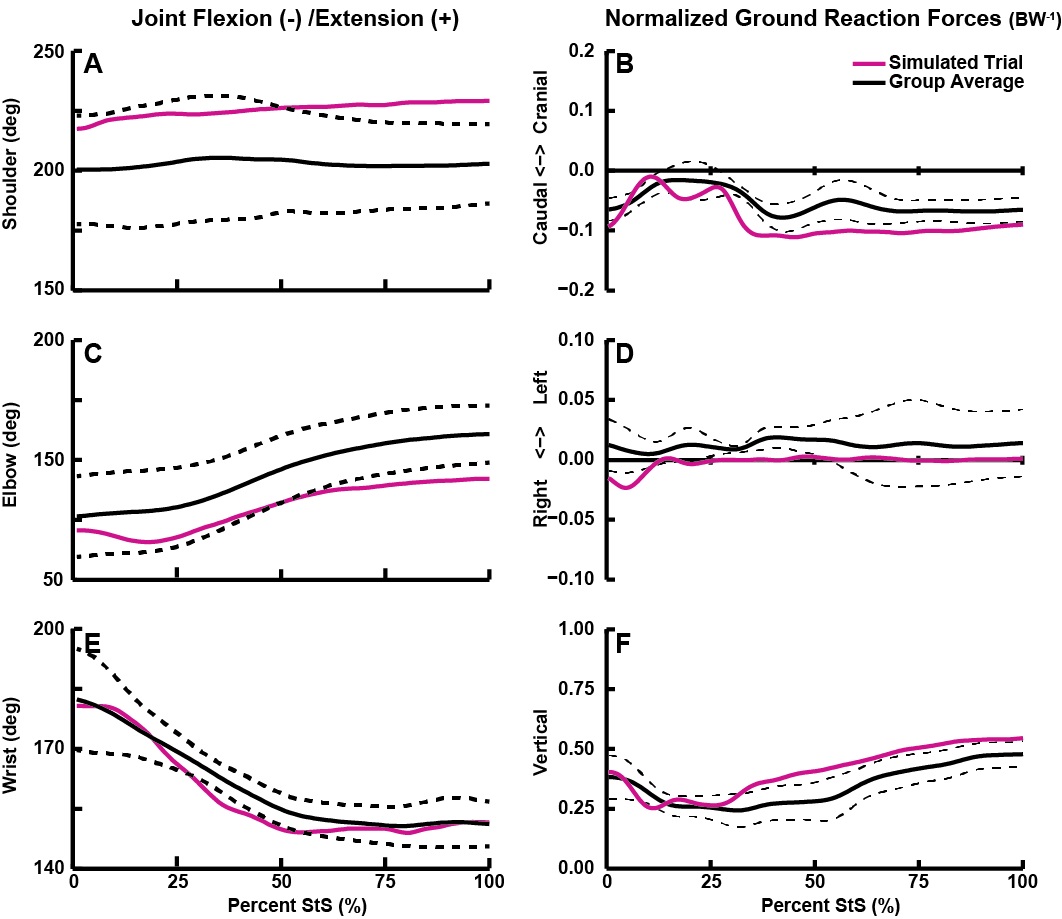

Supplement: Supplementary file 15 [file Image_9.JPEG]

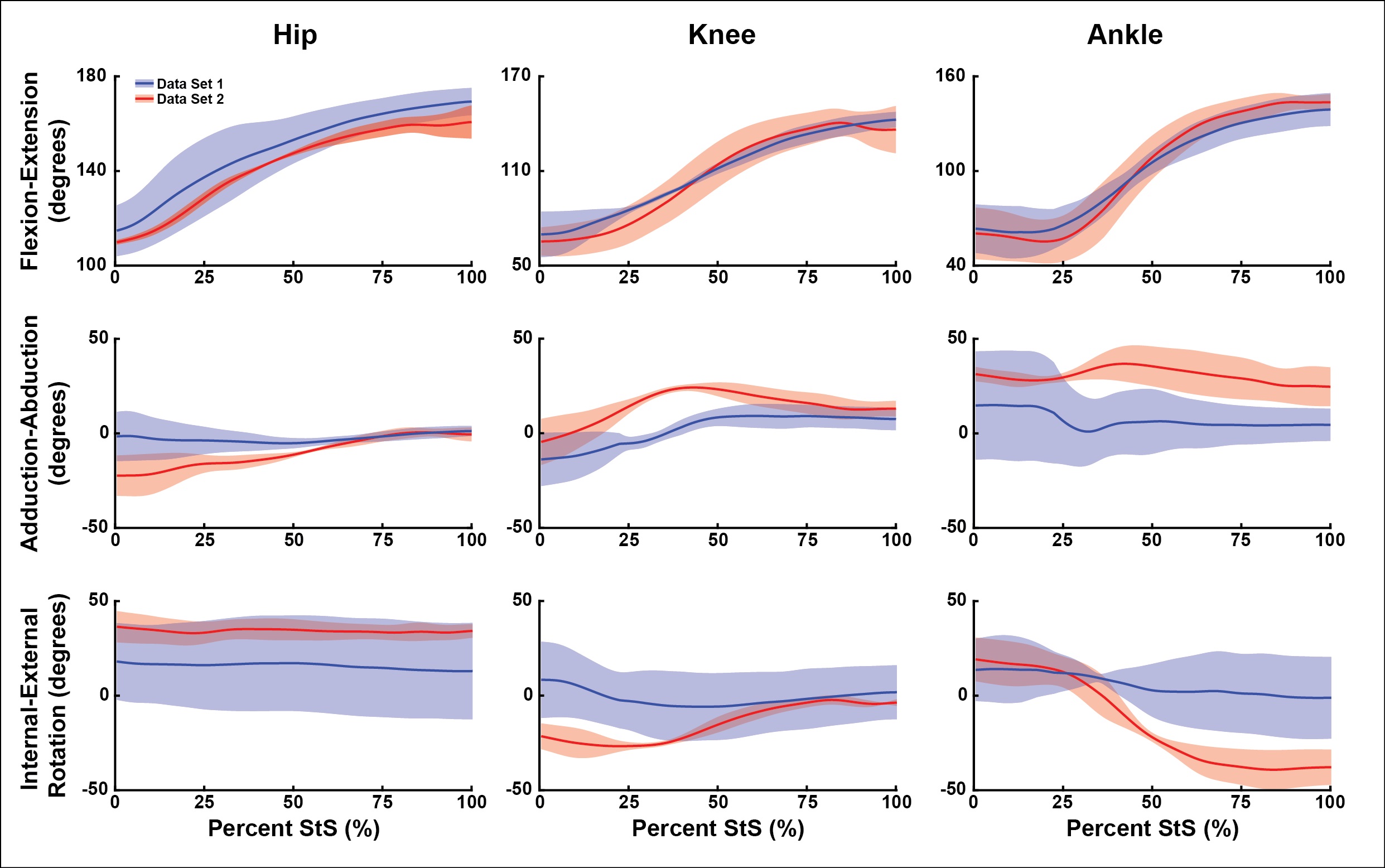

Supplement: Supplementary file 16 [file Image_10.JPEG]
